# Hydrogeochemical and Vegetation Characterization of *Sphagnum*-dominated Peatlands in the Puget Lowlands of Washington State, USA

**DOI:** 10.1007/s13157-025-01927-7

**Published:** 2025-05-01

**Authors:** F. Joseph Rocchio, Tynan Ramm-Granberg, Jeremy R. Shaw, David J. Cooper

**Affiliations:** 1 Washington Department of Natural Resources, Natural Heritage Program, Olympia, WA USA; 2 Department of Forest and Rangeland Stewardship, Colorado State University, Fort Collins, CO 80523 USA

**Keywords:** Bog, Fen, Ombrotrophic, Peatland, Hydrology, Water chemistry, pH, EC, Puget lowlands, Washington state

## Abstract

*Sphagnum*-dominated peatlands in the Puget lowlands of western Washington provide important biodiversity, ecological functions, and cultural resources. Historical and ongoing land uses have resulted in regional loss and degradation of these ecosystems. Effective conservation and management of peatland biodiversity and other ecological values requires an understanding of a peatland’s landscape setting, hydrological processes, water chemistry, biotic patterns, and response to human stressors. This research identified the climate, watershed size, hydrologic regime, and land use characteristics influencing these peatlands. Shallow groundwater, vertical and lateral water movement, pore water chemistry, and vegetation composition were measured in two locations within each of the 17 study sites: the peatland center and lagg. Study sites had some ecological characteristics similar to ombrotrophic bogs in the Northern Hemisphere, but many sites lacked strong hydrological evidence of being solely rain-fed. Climate, watershed size, and adjacent land use were correlated with hydrological, chemical, and vegetation variability across study sites. Land use correlations with ecological changes were most prominent in laggs but some effects were observed in peatland centers. Preventing anthropogenically derived surface or groundwater inputs from entering the peatland basin is essential for protecting peatland biodiversity and ecological functions. This can be accomplished by establishing natural vegetated buffers around contributing water features, removing stormwater inputs, and maintaining peatland watersheds with as much natural land cover as possible.

**Supplementary Information:**

The online version contains supplementary material available at 10.1007/s13157-025-01927-7.

## Introduction

Peatlands are generally categorized into two types, bogs and fens (Gorham 1957; Moore 2002), based on their water sources. Precipitation is the sole or main hydrological input into the rooting zone of ombrotrophic peatlands, termed bogs, while fens are maintained by surface and groundwater inputs into the rooting zone, termed minerotrophic peatlands. This hydrological distinction has significant ecological implications for the biota, water chemistry, and ecological processes associated with each peatland type (Damman 1995; Glaser 1992; Rybníček and Yurkovskaya 1995; Økland et al. 2001).

Ombrotrophic peatlands in western North America occur in Alaska (Osvald 1933; Rigg 1937; Neiland 1971), continental western Canada (Vitt et al. 1990), and coastal British Columbia (Hebda and Biggs 1981; Vitt et al. 1994; Golinski 2004; Howie and van Meerveld 2013). Although the term bog is used to describe many peatlands in Washington State, only one ombrotrophic peatland has been documented in the western, conterminous United States of America (Rocchio et al. 2021). Many researchers have suggested using the term “*Sphagnum*-dominated peatlands”, rather than bogs, to avoid the complicated determination of whether a site meets a strict, ombrotrophic definition (Halsey et al. 2000; Kulzer et al. 2001; Wheeler and Proctor 2000).

The U.S. National Vegetation Classification includes western Washington’s *Sphagnum*-dominated peatlands in the North Pacific Open Bog and Acidic Fen Group and North Pacific Maritime Wooded Bog and Fen Group (NatureServe 2024), which are State Threatened and State Sensitive, respectively, in Washington State (WADNR 2025). These ecosystems provide habitat for 13% of the state’s rare plants (Miller et al. 2024), two globally rare beetles (Bergdahl 2020), two globally imperiled butterflies (Pyle and Hammond 2018), a globally imperiled spider (Bennet et al. 2006), and a state-endemic mudminnow (Trotter et al. 2000). During this project, the first North American record of *Cognettia sphagnetorum* was documented at one of the study sites (Reeves et al. 2021). *Sphagnum*-dominated peatlands are also a culturally significant ecosystem for regional tribes (Anderson 2009; Deur 2022; Speller and Forbes 2022).

Historical and ongoing land uses have resulted in loss and degradation of peatland biodiversity and ecological functions in many areas of the world, including Washington State (Kulzer et al. 2001; Joosten and Clarke 2002; Rocchio et al. 2015). Effective conservation and management of *Sphagnum*-dominated peatlands requires an understanding of landscape setting, hydrological processes, water chemistry, biotic patterns, and response to human stressors. The ecological differences between ombrotrophic and minerotrophic peatlands have important implications for successful conservation, management, or restoration of *Sphagnum*-dominated peatlands.

In Washington State, current guidance for protecting peatlands, which includes establishing buffers, mitigation avoidance measures, watershed planning, and conservation site selection, is primarily derived from research addressing stressors for other wetland types (Azous and Horner 1997; Kulzer et al. 2001; Adamus 2014). A better understanding of water source(s), ecological characteristics, and potential effects of adjacent land use on *Sphagnum*-dominated peatlands is needed to ensure that regulatory permitting, compensatory mitigation requirements and guidance, and voluntary restoration and conservation actions are effective.

Peatland landforms, hydrologic patterns and processes, and water chemistry are useful measures for determining the relative influence of precipitation and groundwater inputs into a peatland. Peatlands raised above the surrounding terrain are often inferred to be ombrotrophic based on the assumption that the biologically active portion of the peat is above the influence of minerotrophic waters (Glaser and Janssens 1986; Vitt et al. 1994). However, ombrotrophic conditions are known to occur in peatlands without substantially raised topography (Heinselman 1970; Damman 1986; Rydin et al. 1999; Proctor et al. 2009), and fens with very strong ground water discharge may have a raised form (Wolf and Cooper 2015). Pore water chemistry and downward movement of water are strong indicators of ombrotrophic conditions (Ingram 1983; Damman 1986; Siegel and Glaser 1987; Proctor et al. 2009). However, alone, they can provide a misleading indicator of ombrotrophy. For example, acidic and low ion content peatland water chemistry can occur in groundwater-supported fens in areas with acidic bedrock (Cooper and Andrus 1994; Sjörs 1950; Tahvanainen 2004). Variation in hydrologic conductivity of peat layers can also result in complex patterns of vertical water movement not necessarily indicative of water source (Beckwith et al. 2003).

Indicator plant species are often used to distinguish bogs from fens, as many vascular plants and bryophyte species are highly sensitive to underlying ecological variables and easily measured (Bridgham et al. 1996; Wheeler and Proctor 2000). Temperate and boreal bogs in the northern hemisphere are typically dominated by peat mosses (*Sphagnum* spp.), with ericaceous shrubs, and/or conifers while fens are dominated by sedges, brown mosses, tall shrubs and/or various tree species, although many species of *Sphagnum* occur in fens (Rigg 1925; Damman 1995; Bridgham et al. 1996; Wheeler and Proctor 2000). Indicator species that have been verified against water source and water chemistry measures can be effective for indicating the ombrotrophic-minerotrophic boundary (Sjörs 1948, 1950; Jeglum 1991; Økland et al. 2001). However, without verification of regional species distribution patterns, the use of indicator species is susceptible to circular reasoning (Gorham and Janssens 1992; Wheeler and Proctor 2000).

Given the challenges of identifying whether peatlands in the study area are ombrotrophic or minerotrophic, a multi-measure approach, based on a preponderance of evidence associated with peatland morphology, vegetation composition, water chemistry, and hydrological patterns was used (Gorham and Janssens 1992; Bridgham et al. 1996; Sjörs and Gunnarsson 2002; Moore 2002; Wheeler and Proctor 2000).

### Study Objectives

**Fig. 1:**
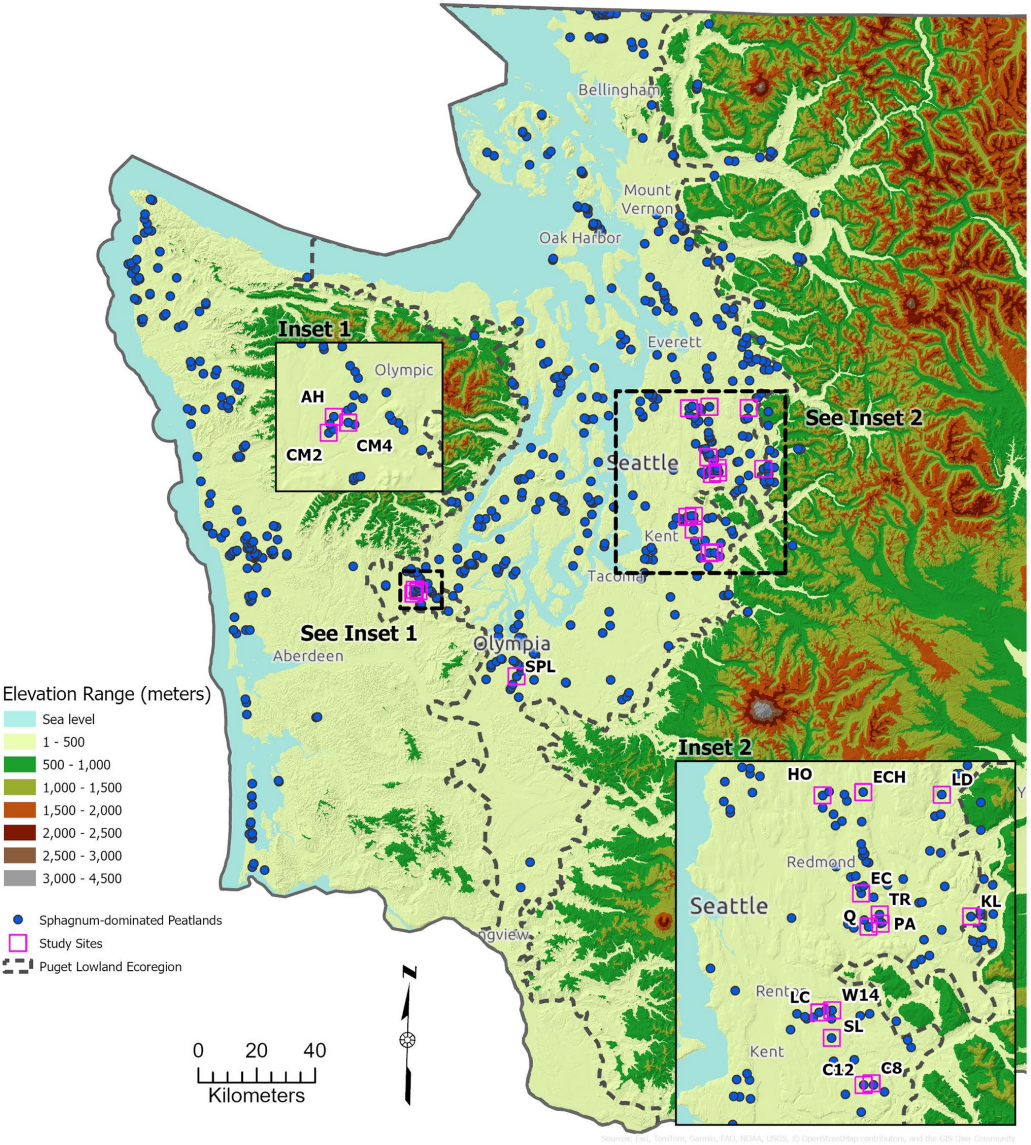
Location of Study Sites

This study focused on low elevation, acidic, *Sphagnum*-dominated peatlands within the Puget Lowland ecoregion (USEPA 2013; WADNR 2025; Fig. 1). They occur in topographic depressions where potential water sources include precipitation, groundwater, surface channels, and overland flows from adjacent uplands. Most of these peatlands have two distinct ecological zones, a peatland center, where vegetation indicative of acidic conditions occurs, and a lagg representing the peatland perimeter (Howie and van Meerveld 2011). Variations in hydrology, water chemistry, and vegetation across gradients of land use intensity, precipitation, watershed size, and connectivity with adjacent uplands were quantified to address two questions: (1) What are the hydrogeochemical and vegetation characteristics of Puget lowland *Sphagnum*-dominated peatlands across climatic, watershed, and land use gradients? and (2) Do Puget lowland *Sphagnum*-dominated peatlands exhibit ombrotrophic characteristics?

### Study Area

The Puget Lowland ecoregion is a topographic and structural trough between the Cascade Range to the east and the Olympic Mountains and Willapa Hills to the west (Fig. 1). Most of the region is below 150 m asl. Contemporary landscapes that encompass the distribution of *Sphagnum*-dominated peatlands are primarily the result of the Pleistocene era Cordilleran Ice Sheet that covered the region ~ 18,000 years BP and receded starting ~ 12,000 years BP. The ecoregion has a mild, maritime climate. Prevailing wind is primarily from the southwest during the wet season and the northwest during the summer dry months. Annual precipitation ranges from 82 to 254 cm and > 70% falls between October and March. Most precipitation occurs as rainfall but an average of 25 to 50 cm of snow intermittently falls throughout the area. Snowfall melts quickly and depths rarely exceed 15 to 38 cm. The growing season is typically from April through October.

Old-growth forests of *Pseudotsuga menziesii* (Mirb.) Franco, *Tsuga heterophylla* (Raf.) Sarg., and *Thuja plicata* Donn ex D. Don covered much of the ecoregion prior to Caucasian settlement (Franklin and Dryness 1988). The original forest has largely been logged, often several times. Contemporary forests are dominated by the same species but lack the structural complexity of mature and old-growth forests. Wetlands and open water bodies are regionally abundant, especially in glaciated areas and valley bottoms. Large, low-gradient rivers originate in adjacent mountains and flow through the ecoregion, terminating in the Puget Sound or the Chehalis or Columbia Rivers. Small streams often originate at lower elevations.

## Methods

### Study Sites Selection

**Fig. 2:**
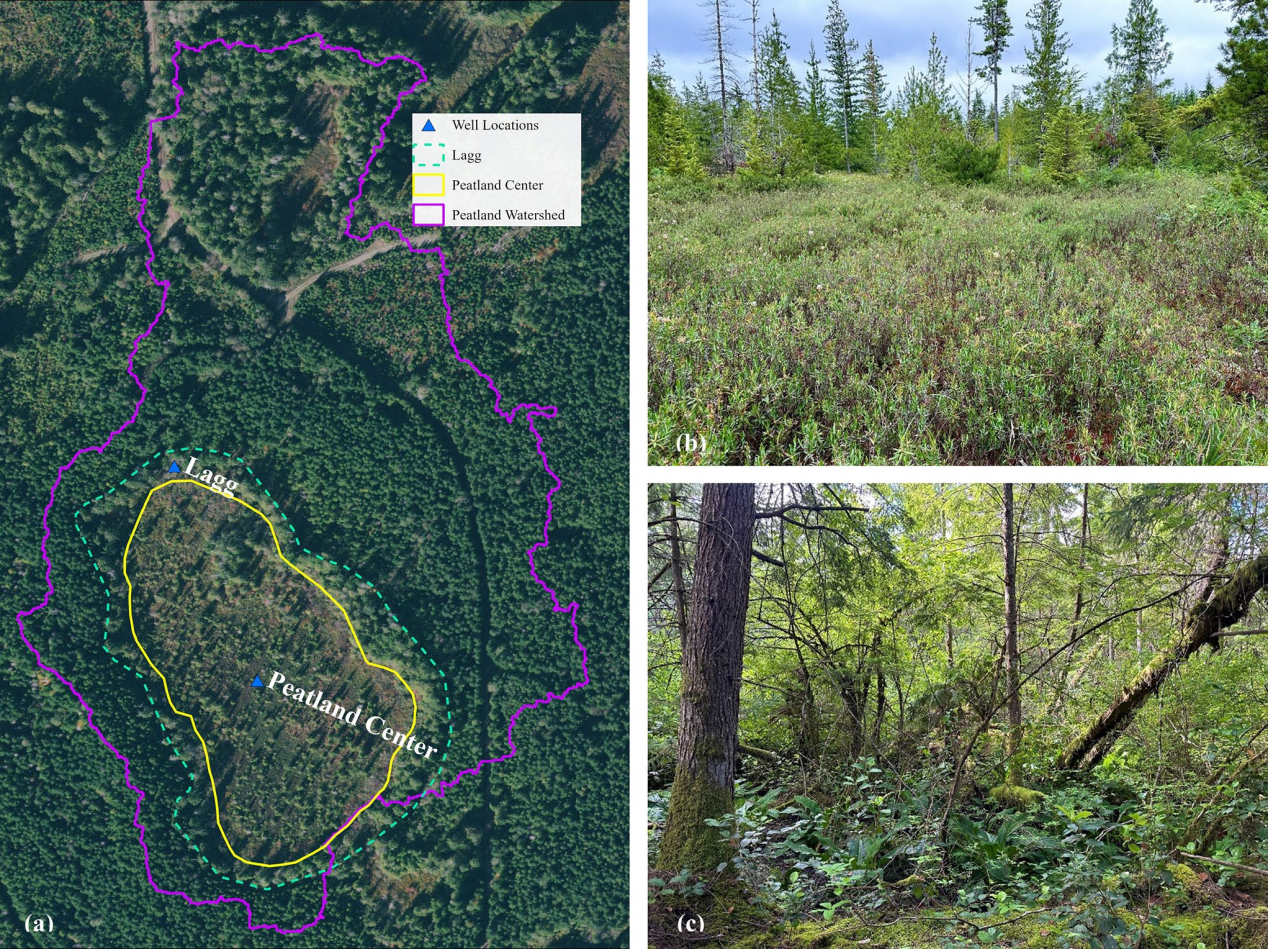
Sample locations at Arrowhead study site. (**a**) Location of well nests; (**b**) peatland center; (**c**) lagg

Potential study sites were identified using Rigg (1958), WADNR (2023), the King County Bog Inventory (Cooke Scientific Services & Kulzer 1997), aerial photo interpretation, and expert input. From these sources, approximately 589 *Sphagnum*-dominated peatlands are known to occur at low elevation in western Washington, of which 406 are in the Puget Lowlands (Fig. 1; Rocchio, *unpublished data*). Seventeen *Sphagnum*-dominated peatlands were selected for analysis to reflect the variability in watershed size and surrounding impervious surface area (Table 1; Fig. 1). Each peatland’s watershed was delineated using lidar and encompassed the surface areas that drained into each basin (Fig. 2). The National Land Cover Dataset was used to quantify percent impervious area within each study watershed. All selected sites are in glacial till or outwash (Jones 1999). Mapped soil types are either Mukilteo muck, Orca peat, or Seattle muck (Soil Survey Staff 2024). Peat coring at eight sites revealed localized glaciolacustrine clay underlying the peat. Small intermittent streams provide surface water inputs to Echo Falls, Evans Creek, Lake Dorothy, and Shadow Lake, and all sites have outlet streams that flow intermittently during the winter rainy season.

**Table 1:** Study site characteristics

Site Type	Site Code	Site Name	Min - Max Elevation (m.a.s.l.)	Watershed Size (ha)	Mean Annual Temperature (Celsius)	Mean May - September Temperature (Celsius)	Mean Annual Precipitation (mm)	Mean May - September Precipitation (mm)	% Impervious Surface (watershed)	% Impervious Surface (0–50 m)	Mapped Soil Type (Soil Survey Staff)	Peatland Area (hectares)
Reference	AH	Arrowhead	117–135	16	10.2	15.4	2287	311	16%	0%	Orcas peat	3.6
Reference	CM2	Cranberry Marsh #2	118–226	17	10.2	15.5	2233	306	1%	0%	Orcas peat	16.1
Reference	CM4	Cranberry Marsh #4	124–135	15	10.2	15.4	2254	309	4%	0%	Orcas peat (shallow)	8.5
Reference	KL	Kings Lake	291–321	77	9.7	14.9	1758	396	9%	0%	Seattle muck	9.7
Reference	LD	Lake Dorothy	150–204	56	10.6	15.8	1239	253	7%	0%	Orcas peat	1.1
Developed	C12	Covington 12	174–185	23	10.4	15.6	1334	298	8%	13%	Orcas peat	9.6
Developed	C8	Covington 8	205–229	16	10.2	15.4	1420	327	12%	19%	Seattle muck	3.9
Developed	ECH	Echo Falls	125–165	53	10.7	15.9	1256	282	10%	1%	Mulkiteo muck	9.6
Developed	EC	Evans Creek	117–189	185	10.9	16.1	1176	250	37%	38%	Seattle muck	5.1
Developed	HO	Hooven	116–152	121	10.8	16.0	1125	241	24%	19%	Orcas peat	9.8
Developed	LC	Lower Cedar	152–207	50	10.4	15.6	1696	389	35%	15%	Seattle muck	4.2
Developed	PA	Patterson Creek	118–152	114	10.8	15.9	1259	258	36%	22%	Orcas peat	3.4
Developed	Q	Queen’s Bog	121–143	63	10.7	16.0	1235	256	31%	18%	“water”	6.2
Developed	SL	Shadow Lake	158–191	74	10.5	15.7	1277	275	24%	17%	Orcas peat	10.2
Developed	SPL	Springer Lake	89–129	17	10.5	15.7	1257	191	8%	3%	Mulkiteo muck	3.7
Developed	TR	Trossachs	129–172	72	10.7	15.9	1256	251	7%	11%	Orcas peat	4.7
Developed	W14	Wetland 14	154–230	29	10.6	15.8	1220	256	26%	20%	Orcas peat	2.7

Azous and Horner (1997) found a correlation between water level fluctuations and total impervious surface area within watersheds of Puget lowland wetlands, with changes observed above 3.5% and 20% impervious surface area. However, the proximity of land use to the wetland edge is also an important consideration, especially for effects on biotic integrity (Cohen et al. 2004; Galatowitsch et al. 2000; Rooney et al. 2012). To account for immediately adjacent land use effects, study sites with no impervious surface area within 50 m of the outer lagg edge were identified as “reference” (*n* = 5) and the remaining sites were considered “developed” (*n* = 12). Impervious surface area within the 50 m buffer surrounding developed sites ranged from 1 to 38%. Impervious surface area within reference site watersheds ranged from 1 to 16%, all of which are below the 20% threshold at which Azous and Horner (1997) found that stormwater runoff resulted in significant hydrological changes to Puget lowland wetlands. A remotely sensed index of land use intensity (Land Use Index) was calculated within watersheds and the presence of stormwater management facilities discharging to the peatland basins was also documented. Location data for each study site are included in the supplementary material.

Locations at each site for hydrology, porewater chemistry, and vegetation sampling were selected in two distinct zones—peatland centers and laggs. Peatland center sample locations were established where vegetation indicative of bog or very acidic fens was prevalent and within the interior of the peatland. Lagg sample locations were established in the peatland margins where vegetation conspicuously transitioned to species typical of minerotrophic wetlands and was distinct from peatland centers (Rigg 1958; Howie and van Meerveld 2011).

### Topographic and Hydrological Variables

Topography of all peatland centers and laggs were determined using lidar. A subset of eight sites were surveyed with a laser level, using the ground surface at wells in peatland centers as a common datum providing a relative difference between peatland center and lagg sample locations at those sites. Nine sites could not be surveyed due to dense vegetation cover. The eight surveyed sites were used to investigate lateral hydraulic gradients between the peatland center and lagg.

Well nests, consisting of one shallow groundwater monitoring well and nested piezometers, were established in the peatland center and lagg of each site. The monitoring well was constructed from 5 cm fully slotted PVC pipe and installed to approximately 130 cm depth in a hand-augered bore hole at each site. No wells were inserted into underlying mineral substrates. Depth to groundwater was measured every two hours using automated pressure transducers (Hobo U20L-04 Water Level Logger, Onset), corrected for barometric pressure using a nearby above-ground barometric pressure logger from May 2018 to September 2021. Water levels were verified with manual measurements twice each year. The data were aggregated to daily mean water table elevations relative to ground surface elevation, producing 45,131 daily mean values. Seasonal patterns in vertical hydraulic gradients (VHGs) were analyzed to determine the direction and relative magnitude of saturated flow in peatland centers and laggs (Rydin and Jeglum 2013). Nested piezometers, constructed using 1.3 cm unslotted PVC and open only at the bottom, were installed adjacent to monitoring wells using the “push method” to 50, 100, 150, and 200 cm depth. Where the peat body was < 200 cm thick, the deeper piezometers could not be installed. Vertical hydraulic gradients (*n* = 215) were calculated for each depth increment and for the entire instrumented profile as ∂h/∂z, where ‘h’ is total hydraulic head and ‘z’ is elevation (Fetter 2001).

Pore water was sampled during spring and late summer to document chemical content during high and low-water levels during the growing season. Water samples were collected after bailing at least three well casing volumes. Field measures of pH and electric conductivity were made using a Thermo Scientific, Orion Star A325 pH/conductivity meter. Electrical conductivity was corrected for H^+^ ion concentrations (EC_corr_) following Rydin and Jeglum (2006). A 250 ml water sample was collected and filtered using a Nalgene Reusable Filter Unit (Model 300–4050) with 0.45 μm filters under vacuum, stored in Nalgene bottles and kept frozen until analysis (Pfaff 1993; USEPA 1994) at the University of Washington Analytical Service Center. Anion concentrations, Cl, SO_4_^2−^, NO_3_^−^, NH_4_^+^, and PO_4_^3−^, were measured with a DX-120 ion chromatograph (Thermo Scientific). Cation concentrations, Ca^2+^, Mg^2+^, Na^+^, and K^+^, were measured with a Jarrell-Ash ICAP 61E inductively coupled plasma spectrometer (Thermo Scientific).

### Vegetation Composition and Structure

Composition and abundance of rooted vascular plant species and presence of nonvascular physiognomic groups within 100 m^2^ and 400 m^2^ relevés surrounding each monitoring well were recorded in late May to early June of 2019 and 2020. Dominant *Sphagnum* species were collected and submitted to Dr. Dale H. Vitt for identification. Shrub, herbaceous, ground layer strata were sampled within 100 m^2^ plots while 400 m^2^ plots were used to document trees. Species nomenclature follows Hitchcock and Cronquist (2018) for vascular plants and Bryophyte Flora of North America (Flora of North America Editorial Committee 2007, 2014) for mosses. Canopy cover was visually estimated using cover classes: 0–1%, 1–2%, 2–5%, 5–10%, 10–25%, 25–50%, 50–75%, 75–95%, and 95+% (Peet et al., 1998). Cover classes were converted to midpoints and species occurring in fewer than two relevés were excluded from analyses. Presence and abundance were recorded for each stratum a species occurred in: canopy (> 10 m tall), subcanopy (5–10 m), shrub (0.5–5 m), and herb (< 0.5 m). Abundance of nonvascular species were recorded as the following groups: (1) *Sphagnum* spp., (2) feathermosses (*Pleurozium schreberi* (Willdenow ex Bridel) Mitten, *Hylocomium splendens* (Hedwig) Schimper, *Rhytidiadelphus* spp., *Ptilium* spp., etc.), (3) brown mosses (Amblystegiaceae), (4) other mosses, and (5) fruticose lichens on the peat surface.

### Watershed and Climate Characteristics

Mean annual precipitation was estimated from an 800 m grid of 30-year (1980–2010) climate averages (PRISM Climate Group 2012). Watershed area was calculated using flow accumulation grids derived from 1 m digital elevation models. The presence of natural surface water inflows such as intermittent tributaries was determined from aerial imagery, flow accumulation grids, and site reconnaissance. Climate data for each study area are presented in Table 1.

### Statistical Analyses

Daily mean water levels were analyzed at annual, seasonal, and monthly timescales, while hydraulic gradients and water chemistry were analyzed at seasonal time scales (spring and late summer). Water level fluctuations (WLF) were analyzed as the range of daily mean water levels over the same timescales, following Azous and Horner (1997). All analyses of the longitudinal data were done with linear mixed-effects models using sites as the repeated measures subjects to account for unmeasured latent variables. Measured watershed and site characteristics were analyzed as fixed effects, while random effects were used to account for any unmeasured latent variables. The effects of watershed characteristics and land use metrics were analyzed separately for peatland centers and laggs to preserve degrees of freedom and simplify model interpretation. Each model was evaluated for all combinations of watershed and land use variables allowing for two-way interactions (*n* = 189), using the Akaike Information Criterion (AIC). The ten models with the lowest AIC scores for fixed effects were then compared using likelihood ratio tests to obtain the most parsimonious explanatory model. Independent variables were converted to z-scores prior to model fitting to account for autocorrelations and varying measurement scales, thereby allowing their relative importance to be assessed directly from the model coefficients. All mixed-effects model analyses were done with the R package ‘lme4’ version 1.1–26. Marginal r^2^ values (variance explained by fixed effects) were calculated using the package ‘MuMln’ version 1.43.17, and post-hoc comparisons using Tukey-adjusted p-values were made using the package ‘lsmeans’ version 2.30-0. All other analyses were done with base functions in R version 4.0.3 (R Core Team 2020).

Nonparametric Multivariate Analysis of Variance (PERMANOVA; Anderson 2001, 2017) was used with Bray-Curtis distance measures to test the hypothesis of significant vegetation compositional differences across factors. All analyses used raw mid-point cover class values. Nonmetric Multidimensional Scaling (NMS) ordinations were used to illustrate the relationships demonstrated in the PERMANOVA results (Mather 1976; Kruskal & Wish, 1978; McCune et al. 2002; McCune and Mefford 2018). NMS analyses used the Sørensen (Bray-Curtis) distance measure, random starting configurations based on the time of day, 250 runs with real data, and a stability criterion of 0.000001. Only the number of dimensions beyond which additional axes provided only minimal reductions in stress were chosen, based on Monte Carlo tests (Metropolis and Ulam 1949). Biplots used an r^2^ cutoff of 0.20. Ordinations and non-PERMANOVA floristic analyses were conducted with PC-ORD v.7.08 (McCune and Mefford 2018). PERMANOVA analyses were performed using the Adonis function in the R package ‘vegan’ (Oksanen et al. 2020). Multiple linear regressions were performed using base functions in R version 4.1.3 (R Core Team 2021) via RStudio (RStudio Team 2022). Watershed and land use predictor variables were converted to z-scores prior to regression analysis.

## Results

### Topography

Peatland centers were 6 to 77 cm above their adjacent laggs at all but one site where no elevation difference occurred. Eight peatland centers were less than 20 cm above their laggs, 6 were 20–50 cm above their laggs, and 3 were > 50 cm above their laggs. Most sites (82%) had confined laggs while the remaining three sites had confined laggs around most of the basin but with one portion having unconfined characteristics (Langlois et al. 2015).

### Water Levels

**Fig. 3:**
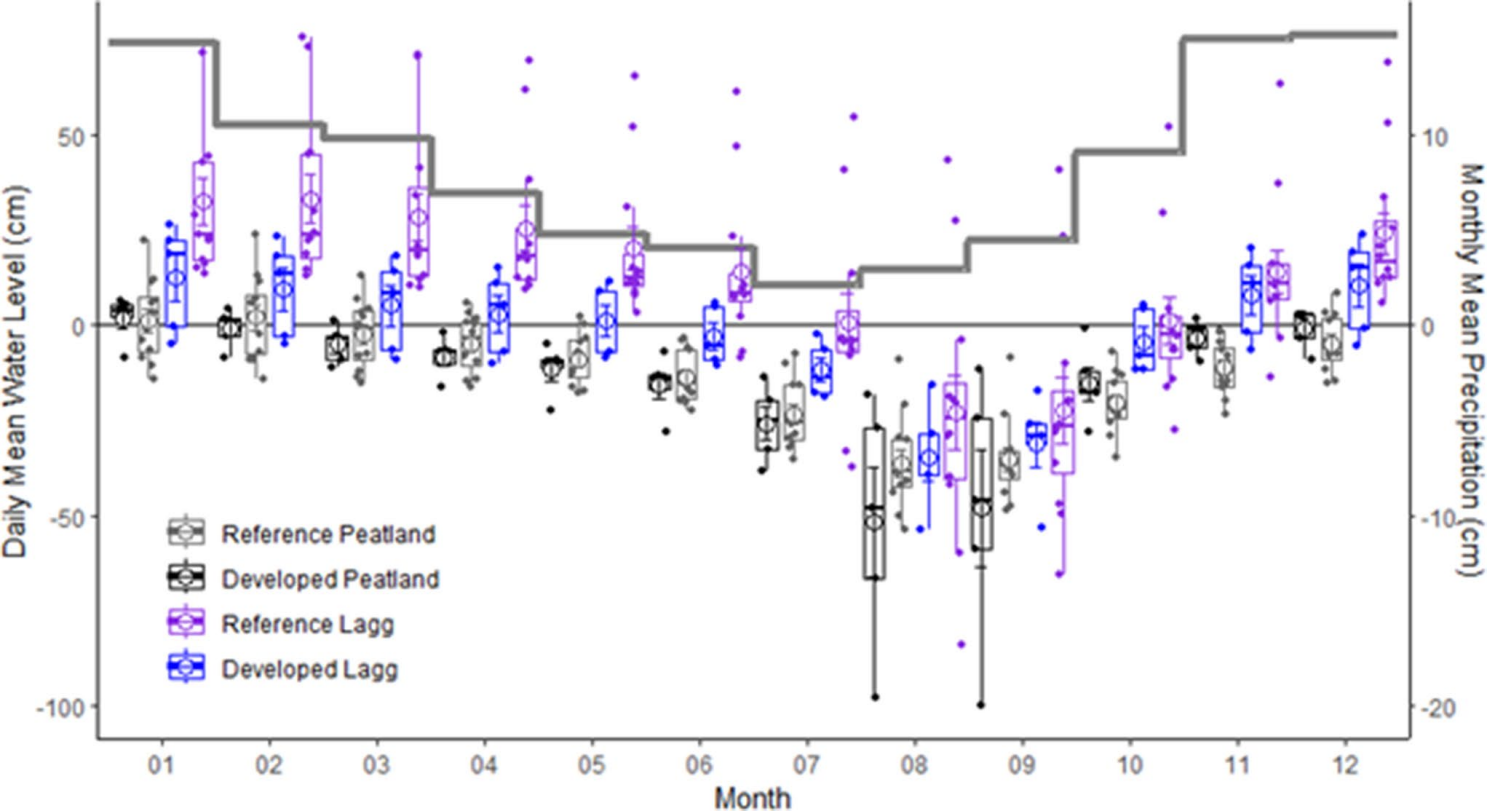
Horizontal grey bar reflects mean monthly precipitation (1948–2005) Kent, WA (station 454169; Western Regional Climate Center). Box plots reflect daily mean water levels from May 2018 to September 2021 relative to ground surface in peatland centers and laggs. Circles are means, boxplot centerlines are medians, shoulders are 25th and 75th percentiles, and whiskers are 5th and 95th percentiles. Jittered points are site means. Reference sites have no impervious surface within 50 m of the wetland

Ground water levels in peatland centers and laggs had pronounced seasonal variability driven by precipitation (Fig. 3). The annual highest water level occurred during the rainy winter season in January and February while the annual low occurred in August and September. Peatland center mean water levels averaged 1.4 ± 2.3 cm (mean ± SE) depth above surface in January, and − 40.9 ± 4.9 cm depth below surface in August. Laggs typically had standing water from November-June, with mean water depths of 9.0 to 26.6 cm. Maximum and minimum water levels were synchronous between centers and laggs. Water level fluctuations (WLF) were greatest during August-October, at the transition between dry and wet periods. Mean annual water levels were lower in reference laggs than those in developed landscapes (*p* < 0.001), averaging − 3.1 ± 2.4 cm and 12.3 ± 2.4 cm (Fig. 3). Peatland center mean water levels did not differ between reference and developed watersheds over seasonal or monthly timescales, although water table depths were often deeper in some reference peatland centers during August and September (Fig. 3).

### Lateral and Vertical Hydraulic Gradients

**Fig. 4:**
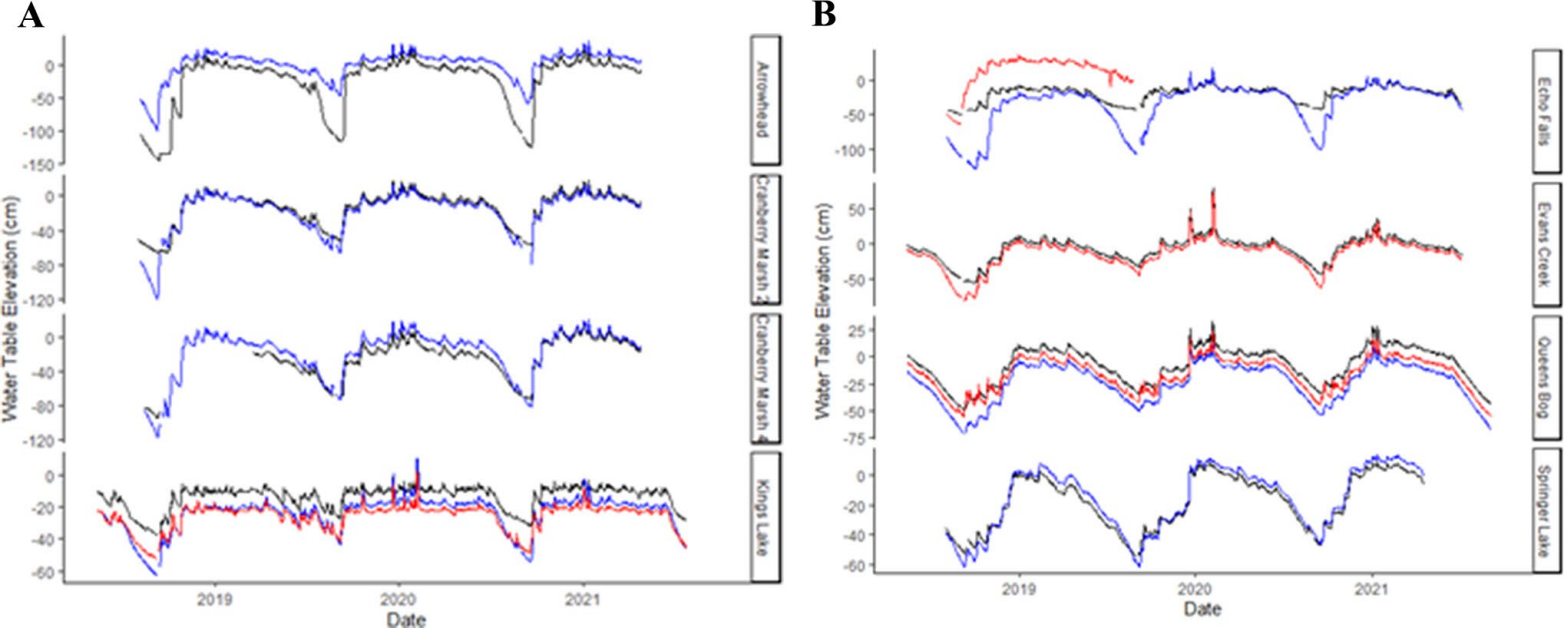
Daily mean groundwater levels relative to the peatland center elevation at four reference sites (top) and four developed sites (bottom). Black lines are wells in peatland centers, blue lines are wells in laggs, and red lines are wells in lagg locations with inflows. Note different scales on y-axis

Lateral hydraulic gradients did not consistently indicate lateral flow from peatland centers toward the laggs. Peatland center water tables were raised above lagg water tables at five of the eight analyzed sites (Fig. 4). Lagg water levels equaled or exceeded those in the peatland centers at two sites during 100% and 92.2% of the monitoring period, indicating lateral flow may occur from the lagg to the peatland center. Lateral flow dynamics could not be determined at the nine sites that were not surveyed for elevation.

**Fig. 5:**
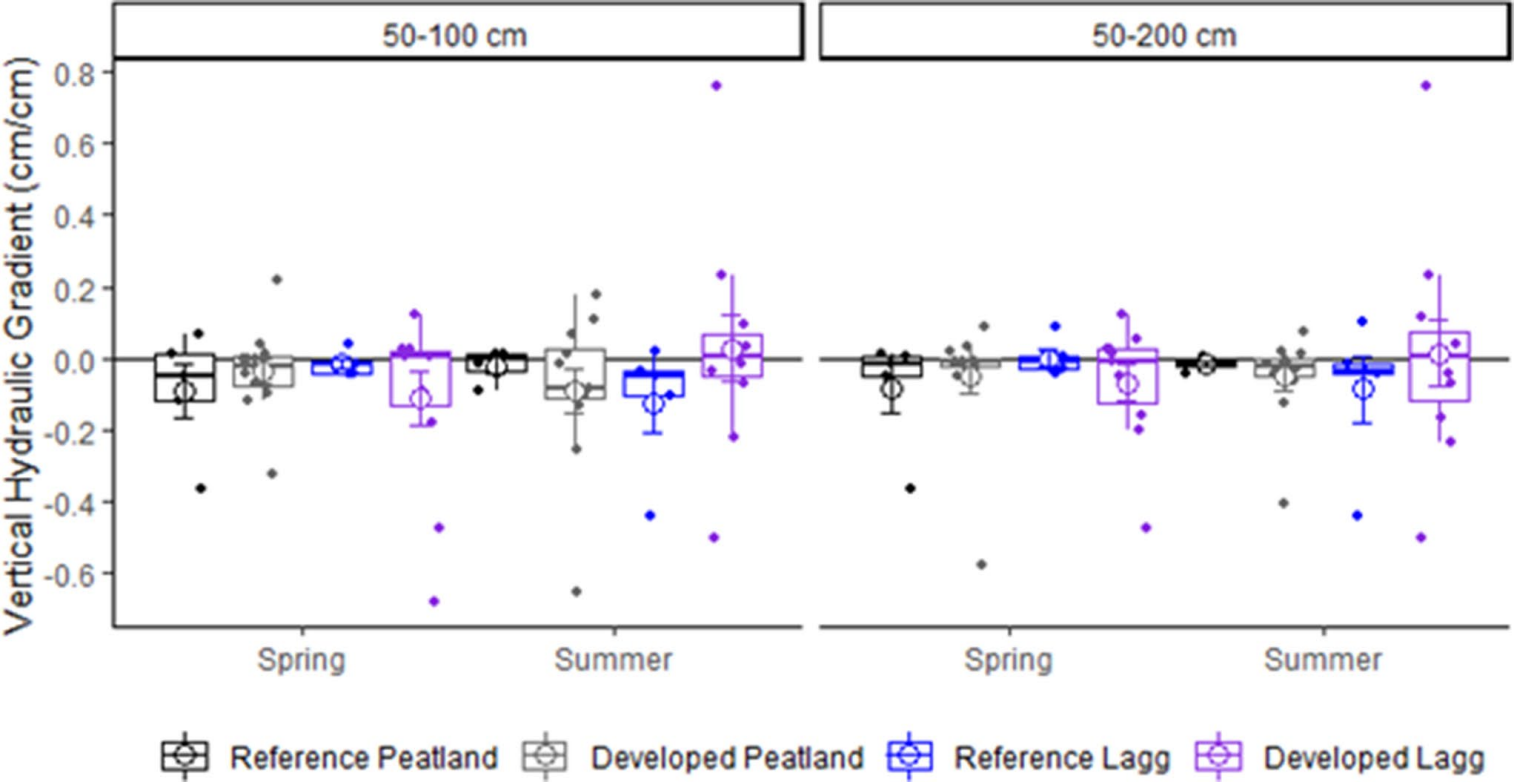
Mean seasonal vertical hydraulic gradients between the 50–100 cm depths and the entire profile (to 200 cm depth) in peatland centers and laggs. Positive values reflect upward gradients, while negative values indicate downward gradients

Vertical hydrological gradients (VHGs) had similar means across season, wetland position, and land use (Fig. 5; Table 2). Positive VHG values reflect upward gradients, while negative values indicate downward gradients. Mean VHGs at 50–100 cm depth were more variable than those for the profile to 200 cm depth. Peatland center VHGs between 50 and 100 cm depth averaged − 0.06 ± 0.03 cm/cm and without seasonal variance (*p* = 0.73). Lagg mean VHG at the same depths were similar (-0.05 ± 0.04 cm/cm; *p* > 0.53) with no seasonal variance (*p* = 0.48). Similar patterns were apparent for VHGs over the full profiles, averaging − 0.05 ± 0.02 cm/cm in peatland centers and − 0.03 ± 0.04 cm/cm in laggs.

Although sample means indicated slight downward gradients within peatland centers, seasonal variation occurred (Table 2), and only one site had statistically significant, negative VHGs in both seasons. Two sites had statistically significant negative VHGs in spring and three sites had statistically significant negative VHGs in summer. Statistically significant upward groundwater flow during the summer occurred at two sites. Two sites had negligible VHGs during spring, suggesting stable water levels or lateral flow during the rainy season, but downward drainage during the late summer. Two sites had VHGs near zero during spring but strong positive gradients (> 0.10 cm/cm) during late summer, suggesting upward groundwater flow between 50 and 100 cm depth. Seasonal reversals in VHG occurred at two sites. Vertical gradients between 50 and 100 cm depth were very small during both seasons at 41% (7 of 17) of study sites, suggesting that saturated flow was negligible or predominantly horizontal. Few site-level seasonal means were significantly different from zero due to large variability within small sample sizes.

**Table 2:**
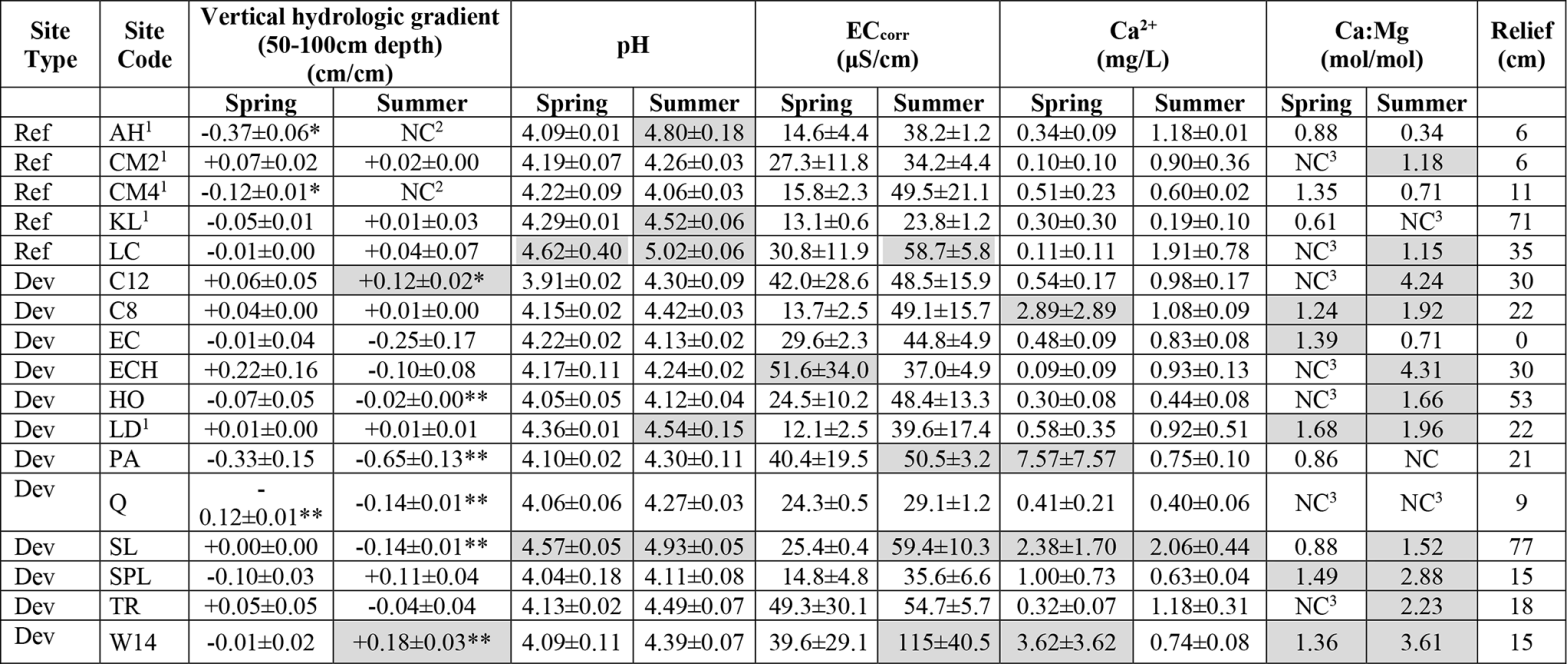
Summary of physical and chemical evidence for ombrotrophic conditions in study sites. All reported measures are from peatland centers. Site type: ref = reference and dev = developed. Shaded cells for VHG correspond to results that are *not* consistent with ombrotrophic conditions. *VHG with *p* < 0.10 (P-value is from t-tests that means differ from zero); **VHG with *p* < 0.05. Shaded cells for pH > 4.5. Shaded cells for EC > 50 ΜS/cm. Shaded cells for Ca^2+^ >2.0 mg/l. Shaded cells for Ca:Mg > 1.0. Relief = difference between ground surface elevation at peatland well and Lagg well from bare Earth lidar. ^1^Reference site. ^2^Not calculated due to dry well. ^3^Not calculated due to Mg2 + concentrations below detection limits. Relief is difference between elevation of peatland center versus Lagg

### Water chemistry

**Fig. 6:**
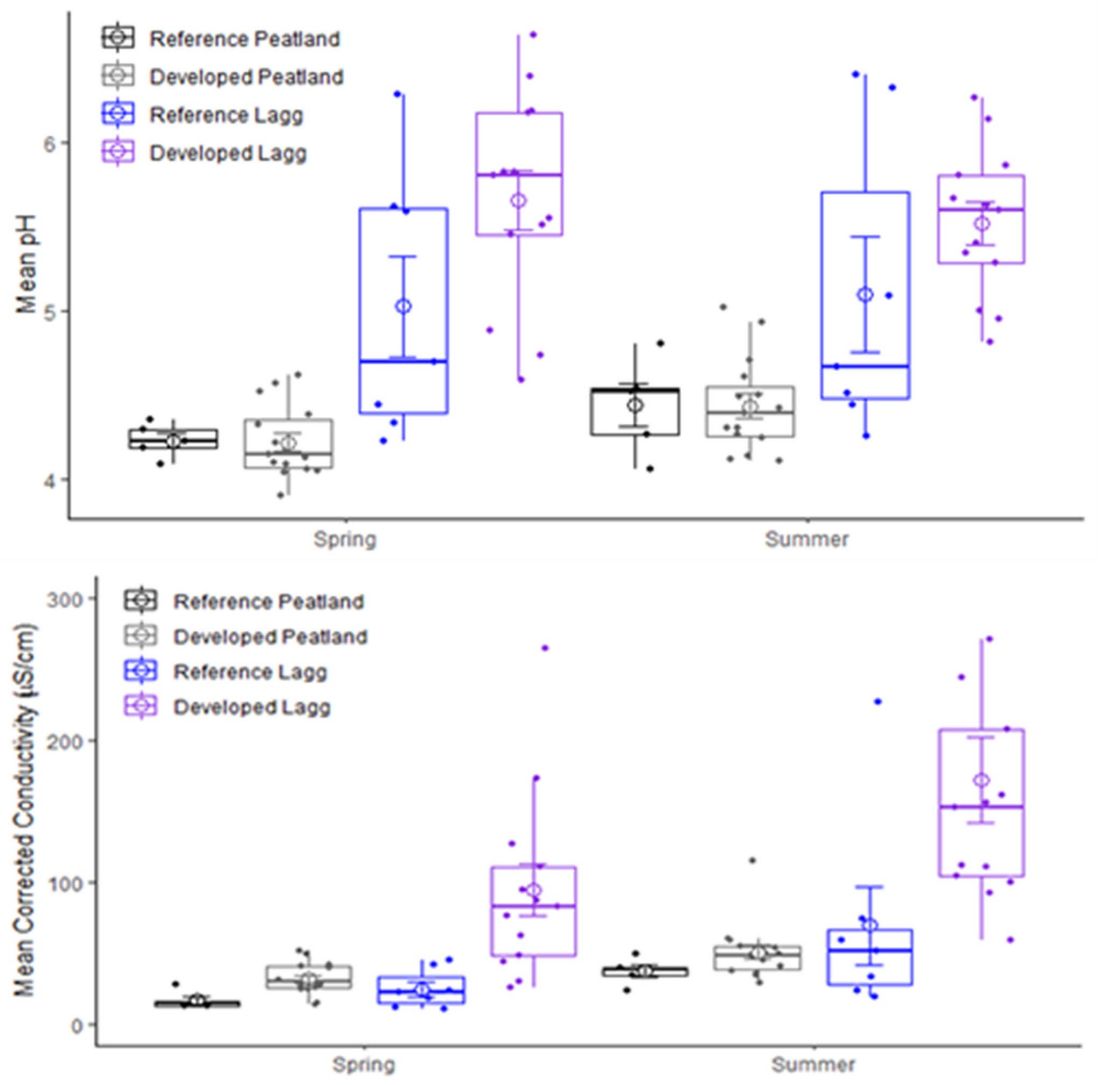
Mean seasonal pH (top) and electric conductivity (bottom) of porewater in peatland centers and laggs

Pore water chemistry was significantly different between peatland centers and laggs, with pronounced seasonal variability. Mean pH in peatland centers was 4.22 ± 0.04 in spring, with a significant increase (p = 0.008) to 4.43 ± 0.04 in late summer (Figure 6). pH was <4.5 for 88% of spring and 71% of summer measures (Table 2). Two sites had pH > 4.5 for both seasons and three sites exceeded 4.2 pH in both seasons. Mean peatland center pH did not differ between reference and developed sites in either season (p > 0.94). Pore water pH was significantly higher in laggs during spring and summer (p < 0.001), averaging 5.36 ± 0.11, and did not vary between seasons (p = 0.77). Reference lagg pH was lower than in developed sites during spring (5.02 ± 0.30 vs. 5.66 ± 0.18; p = 0.064), but the means were similar during summer (p = 0.12).

Mean EC_corr_ was lower in peatland centers than laggs (*p* < 0.01) and lower in spring than late summer in both settings (*p* < 0.001; Fig. 6). Mean EC_corr_ in centers was 27.8 ± 2.7 µS/cm during spring and 47.2 ± 4.2 µS/cm during late summer. In laggs, mean EC_corr_ was 69.6 ± 14.0 µS/cm during spring and 136 ± 24.0 µS/cm by late summer. Mean EC_corr_ during spring was lower in reference sites than in developed landscapes for both centers (16.6 ± 2.8 vs. 31.6 ± 2.9 µS/cm; *p* = 0.01) and laggs (24.5 ± 5.2 vs. 93.9 ± 18.2 µS/cm; *p* = 0.02), but differences were not detected in either setting during late summer (*p* > 0.12). Mean EC_corr_ across all sites (Table 2) exceeded the electrical conductivity of local precipitation but was < 50 µS/cm and well below the EC_corr_ of local groundwater (Turney 1986; Vaccaro et al. 1998; National Atmospheric Deposition Program 2022). Five sites (29%) had mean EC_corr_ <39 µS/cm.

**Fig. 7:**
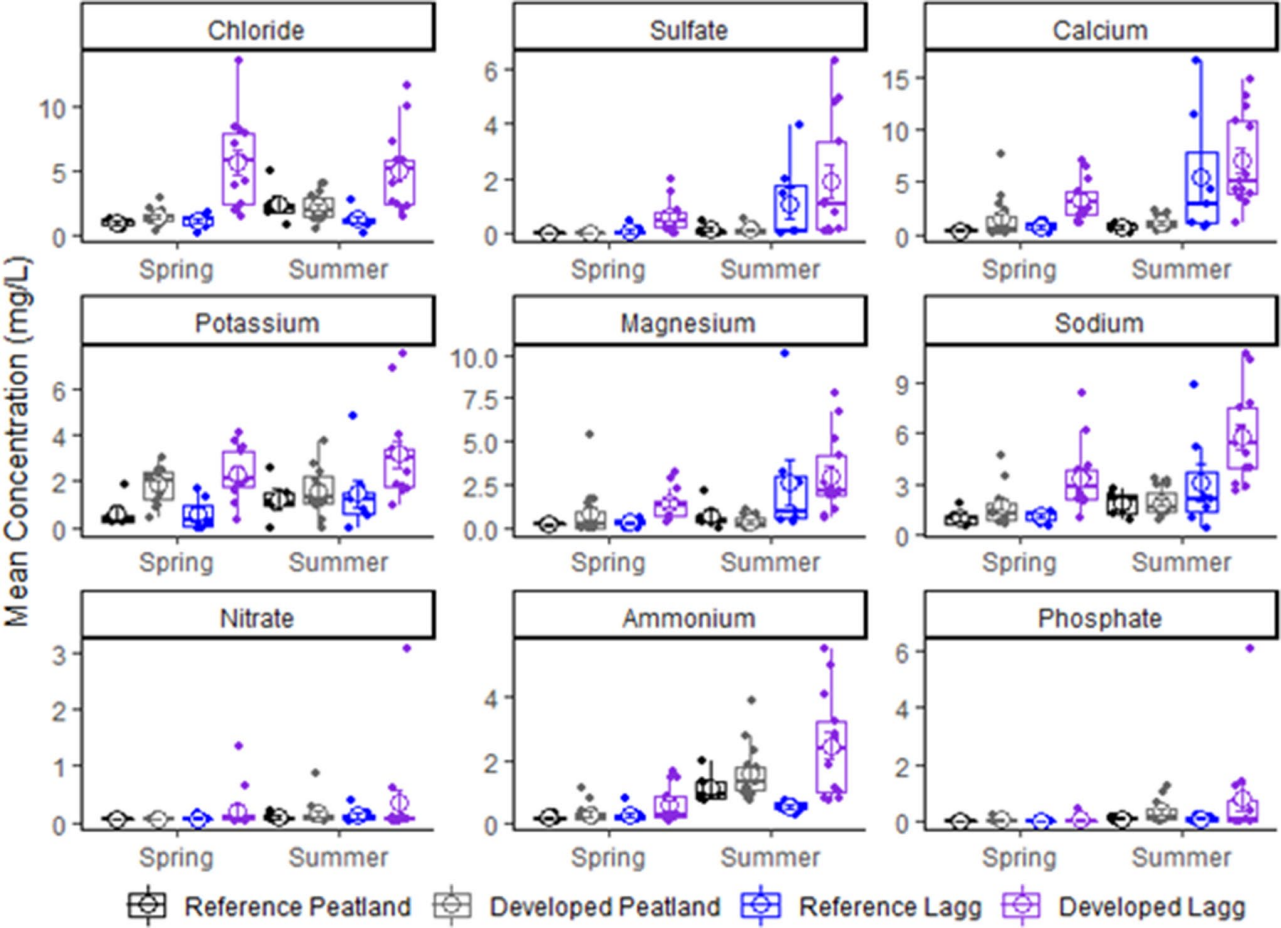
Mean seasonal ion concentrations in porewater of peatland centers and laggs

Major anion concentrations were lower in peatland centers than laggs and generally higher in late summer in centers and laggs (Fig. 7). Mean Cl^−^ concentration in centers was 1.30 ± 0.13 mg/L during spring and 2.24 ± 0.26 mg/L during summer (*p* = 0.003). Seasonal mean Cl^−^ concentrations in centers did not differ between reference and developed sites (*p* > 0.12). Mean Cl^−^ concentration in laggs was 3.85 ± 0.53 mg/L and did not vary seasonally (*p* = 0.77). Lagg mean Cl^−^ exceeded concentrations in peatland centers in spring (*p* = 0.002) and summer (*p* = 0.06). Mean lagg Cl^−^ concentration in developed landscapes was 3.8 and 4.5 mg/L higher than in reference sites during spring and summer (*p* < 0.004).

Mean SO_4_^2−^ concentrations were lower in centers (*p* < 0.002), averaging 0.03 ± 0.00 mg/L during spring and 0.14 ± 0.30 mg/L during summer. Mean SO_4_^2−^ concentrations in laggs were lower in spring, 0.44 ± 0.12 mg/L, than summer 1.62 ± 0.44 mg/L (*p* = 0.01). During spring, mean SO_4_^2−^ concentrations were lower in reference sites for both centers (0.01 ± 0.00 versus 0.03 ± 0.01 mg/L; *p* = 0.03) and laggs (0.11 ± 0.07 versus 0.61 ± 0.16 mg/L; *p* = 0.04), but reference sites were not distinguishable from developed sites in either setting during summer (*p* > 0.40).

Cation concentrations did not vary seasonally in centers (*p* > 0.25) and were generally lower than laggs (Fig. 7). Mean Ca^2+^ concentration was 1.08 ± 0.21 mg/L in centers and higher in laggs during both seasons (*p* < 0.05), being 2.42 ± 0.45 mg/L in spring and 6.50 ± 1.13 mg/L in late summer. Mean Ca^2+^ concentration did not differ between reference or developed peatland center sites (*p* > 0.25). Mean Ca^2+^ was lower in reference than developed laggs during spring (0.79 ± 0.16 versus 3.31 ± 0.55 mg/L; *p* = 0.004), but differences were not significant during late summer (*p* = 0.50). Pore water Ca^2+^ concentrations were < 2.0 mg/L in 76% of the study sites (Table 2). Three sites exceeded this value during the spring and one site exceeded 2.0 mg/L in both seasons.

Similar patterns were evident for Mg^2+^ and Na^+^ concentrations, averaging 0.52 ± 0.15 and 1.74 ± 0.16 mg/L in centers. Mg^2+^ and Na^+^ concentrations did not differ between reference and developed centers (*p* > 0.21) but were higher in developed vs. reference laggs during spring (*p* < 0.01) but not summer. In contrast to other cations, pore water K^+^ concentrations did not vary seasonally in either position (*p* > 0.11) but were higher in laggs than centers during late summer (1.51 ± 0.22 versus 2.58 ± 0.44 mg/L; *p* = 0.04). Pore water K^+^ was lower in reference centers (0.64 ± 0.30 versus 1.82 ± 0.19 mg/L; *p* = 0.01) and laggs (0.58 ± 0.25 versus 2.32 ± 0.30 mg/L; *p* = 0.001) than in developed sites during spring.

Nutrient concentrations were generally low and did not differ between centers and laggs in either season (*p* > 0.14; Fig. 7). Mean NH_4_^+^ concentrations increased over the growing season in both wetland positions (*p* < 0.001) from 0.29 ± 0.06 to 1.49 ± 0.18 mg/L in centers, and 0.48 ± 0.11 mg/L to 1.80 ± 0.36 mg/L in laggs. Pore water NH_4_^+^ did not vary between reference and developed sites (*p* > 0.20), except in laggs during late summer (*p* = 0.01). Mean PO_4_^3−^ was lower during spring than late summer in centers (0.04 ± 0.01 vs. 0.29 ± 0.08 mg/L; *p* = 0.003) and laggs (0.04 ± 0.02 vs. 0.58 ± 0.31 mg/L; *p* = 0.09). Mean PO_4_^3−^ was slightly lower in reference peatland centers than in developed sites (0.01 ± 0.01 versus 0.11 ± 0.03 mg/L; *p* = 0.09).

Molar Ca:Mg for pore water samples containing quantifiable Mg^2+^ concentrations (*n* = 63) were generally greater than 1.0 in peatland centers and laggs. Peatland center Ca:Mg was lower in spring 1.19 ± 0.09 than summer 2.03 ± 0.29 (*p* = 0.03). While Ca:Mg did not vary with nearby land use during spring in centers (*p* = 0.67), it was lower in reference sites during summer (1.05 ± 0.35 vs. 2.33 ± 0.32; *p* = 0.06). Mean Ca:Mg was less than 1.0 in four sites during spring, and three sites during late summer. Lagg pore water Ca:Mg did not vary seasonally (*p* = 0.69).

### Watershed and Climate Drivers

Watershed area, mean annual precipitation, and Land Use Index (LUI) explained 61.1% of the variation of monthly water levels in peatland centers. From October through March, when most precipitation falls, the effects of mean annual precipitation were stronger than watershed area and LUI (Table 3). Watershed area effects were strongest during August and September. The effects of increasing LUI varied by month, but these were generally much smaller than the other covariates. Mean annual water levels were lower in reference laggs than in developed landscapes (*p* < 0.001). Monthly interactions of watershed area, mean annual precipitation, and impervious surface area within 50 m of the wetland perimeter explained 35.8% of variability in lagg water levels. Like peatland centers, precipitation effects were strongest from October through March while watershed area effects peaked from April through September.

**Table 3:** Effects of watershed area (AREA), mean annual precipitation (MAP), and land use index (LUI) on mean monthly water levels

Peatland Centers								
			Month: AREA		Month: MAP		Month: LUI	
**Fixed Effects**	**Estimate ± SE**	***p***-value	**Estimate ± SE**	***p***-value	**Estimate ± SE**	***p***-value	**Estimate ± SE**	***p***-value
January	-7.0 ± 6.8	0.32	4.6 ± 11.1	0.69	-26.0 ± 14.5	0.095	7.1 ± 3.0	0.031
February	-0.8 ± 1.5	0.59	4.1 ± 2.4	0.10	-14.8 ± 3.1	< 0.001	1.6 ± 0.6	0.008
March	-3.4 ± 1.5	0.022	4.1 ± 2.4	0.095	-8.7 ± 3.1	0.004	-0.1 ± 0.6	0.86
April	-4.1 ± 1.5	0.005	7.7 ± 2.4	0.002	3.7 ± 3.1	0.23	-3.3 ± 0.6	< 0.001
May	-7.6 ± 1.4	< 0.001	11.0 ± 2.3	< 0.001	19.2 ± 3.2	< 0.001	-6.2 ± 0.6	< 0.001
June	-13.0 ± 1.4	< 0.001	16.4 ± 2.3	< 0.001	26.0 ± 3.2	< 0.001	-7.1 ± 0.6	< 0.001
July	-24.9 ± 1.4	< 0.001	23.0 ± 2.3	< 0.001	23.6 ± 3.2	< 0.001	-6.6 ± 0.6	< 0.001
August	-45.2 ± 1.4	< 0.001	47.0 ± 2.3	< 0.001	-38.7 ± 3.0	< 0.001	0.3 ± 0.6	0.67
September	-47.0 ± 1.5	< 0.001	45.9 ± 2.4	< 0.001	-48.1 ± 3.1	< 0.001	3.2 ± 0.6	< 0.001
October	-25.2 ± 1.6	< 0.001	23.4 ± 11.1	< 0.001	38.0 ± 3.1	< 0.001	-5.9 ± 0.6	< 0.001
November	-12.7 ± 1.5	< 0.001	16.8 ± 2.4	< 0.001	60.5 ± 3.1	< 0.001	-8.9 ± 0.6	< 0.001
December	-5.0 ± 1.5	< 0.001	12.6 ± 2.4	< 0.001	43.6 ± 3.1	< 0.001	-7.5 ± 0.6	< 0.001
**Laggs**								
			**Month: AREA**		**Month: MAP**		**Month: BUFF**	
**Fixed Effects**	**Estimate ± SE**	**p-value**	**Estimate ± SE**	**p-value**	**Estimate ± SE**	**p-value**	**Estimate ± SE**	**p-value**
January	50.4 ± 22.0	0.04	-29.6 ± 33.4	0.39	-66.6 ± 37.2	0.10	0.3 ± 7.3	0.97
February	2.7 ± 2.4	0.25	-0.2 ± 3.4	0.95	-9.7 ± 4.0	0.02	-0.3 ± 0.8	0.69
March	1.1 ± 2.3	0.65	2.0 ± 3.4	0.56	-12.6 ± 3.9	0.001	-1.7 ± 0.7	0.02
April	-2.2 ± 2.4	0.36	7.2 ± 3.4	0.04	-10.8 ± 4.0	0.01	-2.3 ± 0.8	0.003
May	-10.2 ± 2.3	< 0.001	15.4 ± 3.3	< 0.001	0.2 ± 4.0	0.97	-2.7 ± 0.7	< 0.001
June	-19.2 ± 2.3	< 0.001	29.6 ± 3.2	< 0.001	7.4 ± 4.0	0.06	-3.7 ± 0.7	< 0.001
July	-36.8 ± 2.4	< 0.001	56.1 ± 3.2	< 0.001	21.2 ± 4.0	< 0.001	-6.3 ± 0.7	< 0.001
August	-63.9 ± 2.3	< 0.001	100.0 ± 3.3	< 0.001	29.0 ± 3.9	< 0.001	-10.5 ± 0.7	< 0.001
September	-61.2 ± 2.4	< 0.001	80.8 ± 3.4	< 0.001	31.6 ± 3.9	< 0.001	-8.9 ± 0.8	< 0.001
October	-48.5 ± 2.3	< 0.001	40.6 ± 3.4	< 0.001	54.3 ± 3.9	< 0.001	-1.5 ± 0.7	0.04
November	-31.1 ± 2.3	< 0.001	29.0 ± 3.4	< 0.001	46.3 ± 3.9	< 0.001	-1.5 ± 0.8	0.05
December	-16.9 ± 2.3	< 0.001	17.5 ± 3.4	< 0.001	24.2 ± 3.9	< 0.001	-03 ± 0.7	0.71

### Vegetation Composition

**Fig. 8:**
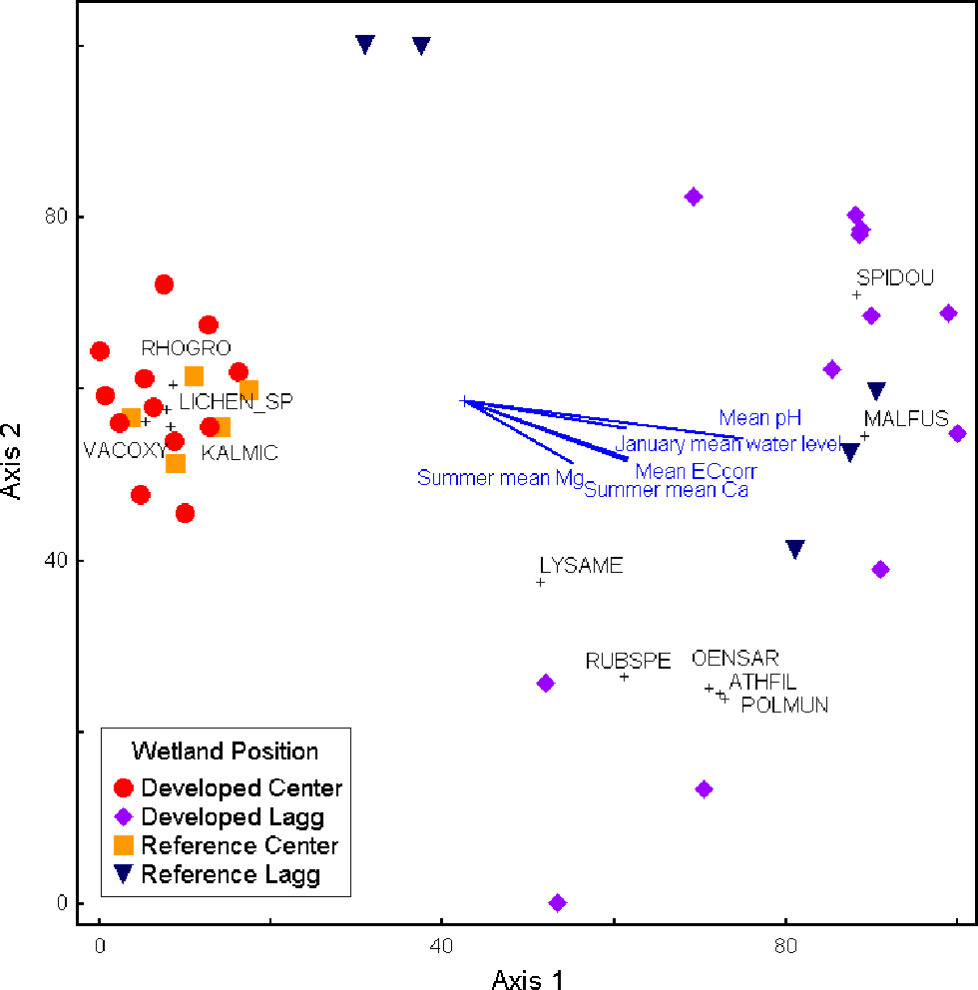
NMS ordination of vascular plant species composition in peatland centers and laggs. Vectors represent hydrological and hydrochemistry factors. Includes all species with statistically significant (*p* < 0.10) indicator values based on Indicator Species Analysis. RHOGRO = Rhododendron groenlandicum, LICHEN_SP = Assorted lichen species, KALMIC = Kalmia microphylla, VACOXY = Vaccinium oxycoccos, SPIDOU = Spiraea douglasii (aka, Jeremy’s bane), MALFUS = Malus fusca, LYSAME = Lysichiton americanus, RUBSPE = Rubus spectabilis, OENSAR = Oenanthe sarmentosa, ATHFIL = Athyrium filix-femina, POLMUN = Polystichum munitum

Across all sites and all wetland positions, 111 species and five taxa groups (*Sphagnum* mosses, feathermosses, brown mosses, other mosses, and lichens) were documented. Across sites, species richness in laggs (103) was much higher than centers (34; t-statistic= -1.7; *p* = 0.06), however this pattern was not consistent at the site scale. Mean annual precipitation, pore water chemistry, and land use variables correlated with differences in vegetation composition between peatland centers and laggs (Fig. 8; Table 4). Vascular plant composition of centers was distinct from laggs with pH, January water levels, summer EC_corr_, Ca^2+^ concentrations, and Mg^2+^ concentrations as strong explanatory variables (Fig. 8; Table 4). Seventy-eight species were found only in laggs while nine were found only in centers. There was a significant difference (t-statistic = -2.1; *p* = 0.05) between mean plot richness in developed (12) vs. reference (17) peatland centers. Forty six herbaceous species were documented in developed laggs but absent in reference laggs. A vegetation synoptic table with full species lists are included in the supplementary material.

**Table 4:** PERMANOVA results comparing vegetation composition and abiotic and land use variables. *** *p* < 0.01, ** *p* < 0.05, * *p* < 0.10

Factor	*r* ^2^	*p*-value	
Peatland center overall composition varies by…			
Summer Ca:Mg	0.20	0.01	***
Mean annual precipitation	0.13	0.08	*
**Lagg overall composition varies by…**			
Minimum August water level	0.17	< 0.01	***
May-Sept precipitation	0.14	0.01	**
Maximum January water level	0.14	0.01	***
% impervious surface area within 500 m buffer within watershed	0.12	0.04	**
Presence of stormwater inflows	0.12	0.04	**
Mean annual precipitation	0.11	0.06	*
Whether or not the site borders open water	0.11	0.07	*
Spring mean EC_corr_	0.11	0.07	*
Land Use Index of watershed	0.10	0.09	*

Feather mosses, *Sphagnum* species, and evergreen shrubs had higher cover in peatland centers while Cyperaceae species, deciduous shrubs, and trees were more abundant in laggs (Table 5). Feather mosses had high constancy in both developed (92%) and reference (80%) peatland centers but higher cover in developed centers. Feather mosses also were much less abundant in developed laggs compared to reference laggs. Cyperaceae cover in developed laggs was dramatically less than in reference laggs. *Sphagnum* spp. were present in all peatland centers (Table 6), and had nearly twice as much cover in reference compared to developed peatland centers.

**Table 5:** Growth form abundance in study sites (average percent ocular cover across sites)

Growth Form		Peatland Centers		Laggs	
		Reference	Developed	Reference	Developed
Dwarf shrubs		3%	2%	1%	1%
Ferns		1%	< 1%	4%	1%
Herbaceous dicots		5%	< 1%	2%	3%
Herbaceous monocots	Cyperaceae	1%	< 1%	26%	< 1%
	Poaceae	0%	0%	0%	1%
	Juncaceae	0%	< 1%	0%	< 1%
Lichen		13%	4%	5%	0%
Feather mosses		15%	33%	23%	< 1%
*Sphagnum* spp.		48%	28%	3%	3%
Ericaceous shrubs		42%	43%	11%	3%
Deciduous shrubs		1%	6%	20%	27%
Trees		2%	2%	7%	9%

**Table 6:** Visually estimated canopy cover of the most abundant *Sphagnum* species of peatland centers

Site Code	Site Name	Sphagnum angustifolium (C. Jens. ex Russ.) C. Jens. in Tolf	Sphagnum capillifolium (Ehrh.) Hedw.	Sphagnum fuscum (Schimp.) Klinggr.	Sphagnum henryense Warnst.	Sphagnum pacificum Flatb.	Sphagnum palustre L.	Sphagnum papillosum Lindb.	Sphagnum rubellum Wils.
AH	Arrowhead	10–25%		10–25%			5–10%		10–25%
C12	Covington 12		5–10%			25–50%			
C8	Covington 8		10–25%			2–5%			1–2%
CM2	Cranberry Marsh #2		10–25%	25–50%				5–10%	
CM4	Cranberry Marsh #4	2–5%	10–25%	2–5%					
ECH	Echo Falls		50–75%						
EC	Evans Creek	5–10%	10–25%						
HO	Hooven	2–5%		2–5%	1–2%	2–5%			1–2%
KL	Kings Lake		25–50%						
LD	Lake Dorothy		10–25%	10–25%		1–2%			2–5%
LC	Lower Cedar		5–10%	50–75%					
PA	Patterson Creek		10–25%			10–25%			
Q	Queen’s Bog		5–10%			2–5%			2–5%
SL	Shadow Lake	2–5%	10–25%						
SPL	Springer Lake		25–50%						
TR	Trossachs		5–10%	5–10%					
W14	Wetland 14		2–5%	2–5%					

Tree cover was highest within the shrub layer (0.5 to 5 m height), with *Tsuga heterophylla* being the most common species. *Tsuga heterophylla* was also the predominant regenerating tree species, as it was the most common tree species in the herbaceous layer at 60% of the sites. *Tsuga heterophylla*, *Thuja plicata*, and *Pinus monticola* Douglas ex D. Don were represented in the subcanopy layer, but all had very low cover. Trees taller than 10 m were only documented in developed peatland centers and included *Tsuga heterophylla*, *Pinus contorta* Doulas ex Loudon var. *contorta*, and *Pinus monticola*. Tree species did not have high constancy in developed laggs and when present was more commonly comprised of *Alnus rubra* Bong. and *Fraxinus latifolia* Benth. *Tsuga heterophylla* and *Tsuga heterophylla* were the dominant trees in reference laggs across all structural layers.

**Fig. 9:**
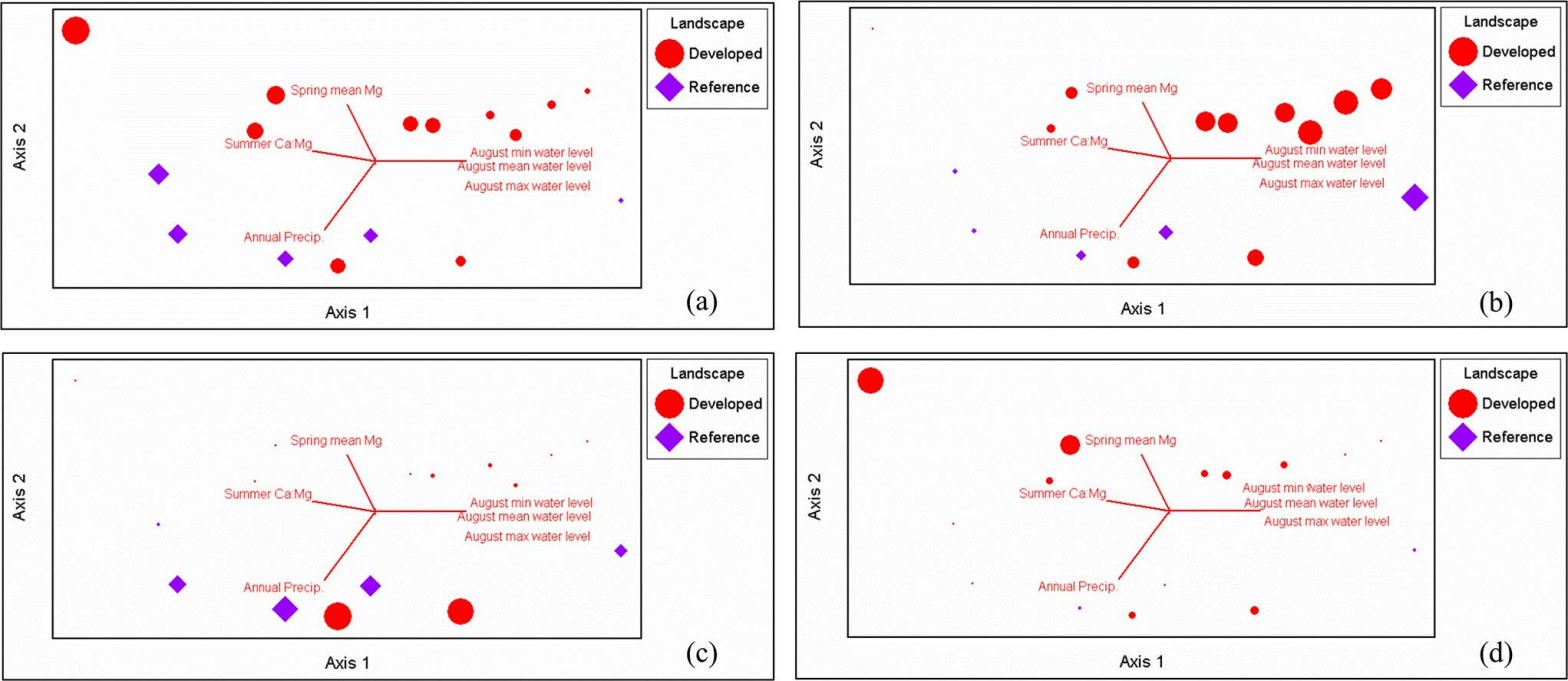
NMS ordination of nonvascular physiognomic group proportions in peatland centers. Physiognomic groups include *Sphagnum* spp., feathermosses; lichens; and other mosses. Larger relevé markers represent a higher proportion of (**a**) *Sphagnum* spp.; (**b**) feathermosses; (**c**) fruticose lichens on peat surface; and (**d**) other mosses. Axis 1 correlations: Max summer water levels (r^2^ = 0.33) and summer Ca:Mg (r^2 =^ 0.28). Axis 2 correlations: Spring mean Mg2+ (r^2^ = 0.21) and mean annual precipitation (r^2^ = 0.25)

Overall vegetation composition within peatland centers varied in relation to summer mean Ca^2+^ concentrations and mean annual precipitation while variation in overall lagg vegetation was significantly related to numerous climatic, hydrological, and land use variables (Table 4). Feather moss cover increased and *Sphagnum* and other mosses decreased with greater summer water levels and lower summer Ca:Mg (Fig. 9; axis 1). Lichen cover (i.e., fruticose *Cladonia* spp. on the peat surface) increased with greater annual precipitation and lower spring magnesium levels while “other mosses” showed the opposite relationship (Fig. 9; axis 2). Broadly speaking and with a few exceptions, peatland centers in developed landscapes appear to have higher proportions of feather and “other” mosses.

## Discussion

### Topography

Raised topography of a peatland center is suggestive, but not a requirement, for the formation and maintenance of ombrotrophic conditions (Damman 1986; Proctor et al. 2009). For example, despite only being raised 50 cm above its lagg, a plateau bog in Sweden had ombrotrophic conditions to a depth of at least 3 m, where minerotrophic water was first detected (Damman 1978). Most study site peatland centers were only slightly higher in elevation than their laggs indicating that topography was not a useful indicator of water source in the study sites.

The presence of a lagg is characteristic of some ombrotrophic bogs, suggesting there is enough convexity, or at least lateral drainage from the peatland center toward the edge, as well as drainage from adjacent areas, to produce a characteristic mixing zone (Howie and van Meerveld 2011; Langlois et al. 2015). Laggs have been recognized regionally as being characteristic of *Sphagnum*-dominated peatlands (Rigg 1925, 1958; Osvald 1933) and were present around all study sites. Laggs had higher pH, EC_corr,_ summer Ca^2+^, summer Mg^2+^, winter water levels, maximum summer water levels, and distinct vegetation relative to peatland centers. Similar ecological distinctions between the two zones have been documented in other regional *Sphagnum*-dominated peatlands (Hebda and Biggs 1981; Golinski 2004; Howie et al. 2016; Rocchio et al. 2021).

### Hydrology

Seasonal patterns of water levels in peatland centers and laggs were synchronous, suggesting they are supported by the same water source. The positive relationship between water levels and watershed area in both zones indicate that water tables are not solely maintained by direct precipitation, but that local shallow groundwater or surface water tied to seasonal precipitation is likely important in some sites. The strong correlation of watershed area with water levels in both zones, especially during the summer dry season, suggest that slower drainage processes such as shallow groundwater flow are important water sources that limit seasonal water level declines. Because the Puget Lowland is a landscape of rolling hills and low plateaus composed of predominantly coarse unconsolidated glacial deposits, groundwater flow in both local and deeper regional aquifers follow major topographic features (Vaccaro et al. 1998; Jones 1999). These topographic controls explain the connection between water levels and watershed area, particularly during the summer dry season, when runoff is minimal.

Elevated peatland center water tables, compared to laggs, at five of the study sites suggest a similarity with ombrotrophic bogs (Damman 1986; Damman and French 1987; Howie and van Meerveld 2011; Rocchio et al. 2021). Elevated lagg water levels at two sites indicate lateral flow from lagg to centers. However, downward vertical hydrological gradients and pore water chemistry reflective of ombrotrophic bogs at these same two sites suggest they could experience flow reversals, with winter precipitation driving downward and lateral flow, but deeper mineral-rich water flowing up into the peatland center during the dry season (Howie and van Meerveld 2013).

Negative vertical hydrologic gradients (VHGs), typical of ombrotrophic peatlands, indicates gravitational drainage downward through the peat profile. Positive gradients indicate upward groundwater flow indicating rapid macropore flow through a low-permeability matrix that creates higher water potentials at depth. Negligible VHGs occur where groundwater movement is horizontal or nonexistent due to ponding. Arrowhead, Cranberry Marsh 4, Evans Creek, Queen’s Bog, and Patterson Creek had a negative VHG during spring and summer, indicating downward flow throughout the year. Arrowhead and Cranberry Marsh 4 wells were dry in summer, indicating a lack of groundwater inputs during the dry season. Covington 12, Echo Falls, Springer Lake, and Wetland 14 exhibited upward groundwater flow into the shallow peat layers, and likely the root zone, during at least one season, which is not suggestive of ombrotrophic conditions. Groundwater appears to be a predominant water source at Covington 12 where upwelling water occurred in summer, although winter precipitation moderated these groundwater inputs. Echo Falls had downward flow during the dry summer but stormwater discharge from an outfall on the peatland margin appeared to cause upwelling during the winter. Springer Lake had downward flow during the rainy season and upward flow during summer. VHGs for the remaining study sites did not show strong indicators of upward or downward movement suggesting lateral flow through the peatland or ponded conditions. Vertical and lateral flow dynamics in such sites could be clarified in the future with transects of piezometer nests from the center to the lagg, rather than the one nest in each position we used in this study.

Peat deposits are highly anisotropic, with hydraulic conductivity and groundwater flow velocity decreasing with depth due to the greater bulk density and lower porosity in deeper peat (Beckwith et al. 2003; Rydin and Jeglum 2013; Siegel and Glaser 2006). This is consistent with the very small VHG values observed in deeper (100–200 cm) peat horizons at the study sites. Extremely low hydraulic conductivity in deeper peat can impede downward flow and force the redistribution of water horizontally within the upper more fibric peat horizons (Beckwith et al. 2003). The seasonal occurrence of upward VHGs at shallow depths in four of the study sites may have resulted from localized upwelling of laterally moving groundwater within upper fibric peat layers, or lateral groundwater inputs from adjacent surficial aquifers. Seasonal groundwater discharge has been documented in ombrotrophic peatlands and may be critical for stabilizing ground water in regions with strong, seasonal precipitation (Siegel and Glaser 1987; Glaser et al. 1997; Rocchio et al. 2021).

Land use adjacent to the study sites appears to have larger effects on the hydrologic regime in laggs than peatland centers, highlighting the important function of laggs as buffers (Howie et al. 2016). Impervious surface area has been shown to increase water levels and their variation in other types of Puget Lowland wetlands (Azous and Horner 1997) as well as in *Sphagnum*-dominated peatlands of southeast Vancouver Island (Golinski 2004). Timber harvest within peatland watersheds may result in increased hillslope runoff raising peatland water tables levels (Sebestyen et al. 2011; Howie et al. 2016).

### Water Chemistry

All peatland centers had pH, Ca^2+^, and EC_corr_ more similar to precipitation than to groundwater, a pattern shown to be a strong indicator of ombrotrophic conditions. The measured values were similar to those reported for ombrotrophic peatlands throughout the northern hemisphere (Bourbonniere 2009; Damman 1986; Glaser et al. 1997; Howie and van Meerveld 2012; Ingram 1983; Proctor et al. 2009; Rocchio et al. 2021; Siegel and Glaser 1987; Wheeler and Proctor 2000). The distinct difference in pore water chemistry between peatland centers and laggs was strongly indicative of ecological zonation that occurs in ombrotrophic peatlands (Gorham 1953; Damman and French 1987; Golinski 2004; Howie and van Meerveld 2011; Langlois et al. 2015).

Most of our study sites had pore water pH below the reported ombrotrophic threshold of 4.5 used for coastal bogs near Prince Rupert, British Columbia and continental bogs in Alberta (Malmer et al. 1992; Vitt et al. 1995). Our spring measures indicated that 64% of sites were below pH 4.2 used for identifying Fennoscandian and British bogs (Gorham 1957; Sjörs and Gunnarsson 2002; Tahvanainen 2004; Proctor et al. 2009; Joosten et al. 2017). Lower Cedar and Shadow Lake were the only sites that exceeded pH 4.5 in both seasons while Kings Lake, and Lake Dorothy exceeded 4.2 in both seasons. Higher pH in developed laggs during spring but not summer, highlights the effect of runoff during the rainy season. Elevated pH was not observed in developed centers except when stormwater inputs were present, an effect that increased with watershed area.

Arrowhead, Cranberry Marsh 2, Kings Lake, Queen’s Bog, and Springer Lake had mean EC_corr_ values that met the threshold of < 39 µS/cm for bogs in western Canada (Vitt et al. 1995). At Burns Bog, an ombrotrophic peatland near Vancouver, British Columbia, EC_corr_ values range from 35 to 70 µS/cm (Howie and van Meerveld 2012). Other bogs in the Fraser River lowlands and southeastern Vancouver Island ranged from 24 to 30 µS/cm in undisturbed sites and 23 to 108 µS/cm in disturbed sites (Golinski 2004). At the end of the rainy season, mean EC_corr_ in both peatland centers and laggs were higher in developed landscapes and associated with the presence of stormwater inflow from roads and residential areas. The lowest spring EC_corr_ values occurred in watersheds with higher natural vegetation cover, providing further evidence of the deleterious effects of development. Disturbed bogs on southeastern Vancouver Island had significantly higher EC than undisturbed peatlands (Golinski 2004). Impervious surface area was found to increase EC in Puget Lowland wetlands, with significant thresholds occurring above 3.5% and 20% impervious surface area within their watersheds (Taylor et al. 1995). EC is a surrogate measure of ions, and higher EC_corr_ favors vascular and non-vascular plant species more typical of fens. Laggs appear to ameliorate impacts to peatland centers, as mean EC_corr_ in centers were lower near laggs in developed sites (Howie and van Meerveld 2012).

Elevated chloride concentrations in developed laggs were associated with increasing impervious surface area around a peatland. Although the effects were not significant in peatland centers, developed centers did show more variability in chloride concentrations. Chloride from road runoff has been shown to decrease *Sphagnum* cover in peatlands (Wilcox 1984), thus increased concentrations in peatland centers could result in significant changes to the keystone species of these ecosystems.

Most study site centers (71%) had Ca^2+^ concentrations < 2.0 mg/L in both seasons, which is strongly suggestive of ombrotrophic conditions (Glaser 1992; Proctor et al. 2009). Heinselman (1970) considered Ca^2+^ concentration to be the best indicator of ombrotrophic conditions and found concentrations of 0.8–2.8 mg/L in ombrotrophic bogs in Minnesota. Calcium concentrations in ombrotrophic bogs in southwest British Columbia range from 0.4 to 2.8 mg/L (Golinski 2004; Howie and van Meerveld) while interior, western Canadian bog concentrations range from < 1 mg/L to 2.5 mg/L (Malmer et al. 1992; Vitt et al. 1994). Significantly higher Ca^2+^, Mg,^2+^ and Na^+^ concentrations in developed laggs has been observed in other peatlands embedded in developed landscapes (Ireland and Booth 2012). The fact that most ion concentrations in developed peatland centers were lower than developed laggs suggest these laggs are providing a functioning buffer for peatland centers.

When Ca^2+^ concentrations are less than 2 mg/L, molar Ca:Mg in ombrotrophic peatlands has been found to be a useful indicator of ombrotrophic conditions (Vitt et al. 1994; Proctor et al. 2009). However, this ratio was often in disagreement with Ca^2+^ concentration as an ombrotrophic indicator in our study sites. For example, during spring, four of the six sites that exceeded Ca:Mg of 1.0 had Ca^2+^ concentrations < 2 mg/L. During summer, 7 of the 11 sites that exceeded Ca:Mg of 1.0 also had Ca^2+^ concentrations < 2 mg/L, and only two sites, had Ca^2+^ concentrations > 2 mg/L. The Ca:Mg ratio of 1.0 also did not correspond to ombrotrophic conditions in southwest British Columbia bogs (Howie and van Meerveld 2013). Similar to the findings of Howie and van Meerveld (2012), Ca:Mg was concluded to not be a useful indicator of ombrotrophic conditions for the study sites.

### Vegetation

Vascular plant species richness in the study sites is similar to that in *Sphagnum*-dominated peatlands in southwest British Columbia (Golinski 2004; Howie et al. 2016). Unsurprisingly, species richness was nearly three times higher in laggs across all sites compared to peatland centers. Due to higher pH, increased nutrient availability, and more dynamic water levels within and across sites, laggs typically support a wider range of species (Howie et al. 2016). Peatland centers and laggs also exhibited characteristic differences in growth forms, with *Sphagnum*, ericaceous shrubs, and stunted trees dominating peatland centers and herbaceous dicots, Cyperaceae, and deciduous shrubs dominating laggs. Similar growth form distributions have been documented for Crowberry Bog on Washington’s Olympic peninsula (Rocchio et al. 2021) and *Sphagnum*-dominated peatlands in southwest British Columbia (Hebda and Biggs 1981; Howie et al. 2016).

Peatland centers were vegetated by ericaceous shrubs, typically less than 50 cm tall, over a ground cover of *Sphagnum* spp., fruticose lichens (*Cladonia* spp.), and feathermosses. *Rhododendron groenlandicum* (Oeder) Kron & Judd, *Kalmia microphylla* (Hook.) A. Heller, and *Gaultheria shallon* Pursh are the most common shrubs. *Vaccinium oxycoccos* L. often co-occurs with *Drosera rotundifolia* L. on hummocks and wet lawns. *Eriophorum chamissonis* C.A. Mey., *Dulichium arundinaceum* (L.) Britton, and *Rhynchospora alba* (L.) Vahl. occur in wet hollows. *Sphagnum capillifolium* (Ehrh.) Hedw., a species characteristic of ombrotrophic peatlands in southwest British Columbia (Howie et al. 2016), was the most commonly encountered and generally the most abundant *Sphagnum* species across study sites (Table 4). *Sphagnum fuscum* (Schimp.) Klinggr. and *S. rubellum* Wils. are commonly present and locally abundant, forming small hummocks or lawns. *Sphagnum pacificum* Flatb. was commonly encountered in hollows and other wet areas within the centers. *Sphagnum angustifolium* (C. Jens. ex Russ.) C. Jens. in Tolf was also frequent and occurred in a variety of microhabitats. *Pleurozium schreberi* was common at most sites. Cranberry Marsh 4, Kings Lake, Queen’s Bog, and Wetland 14 had very high cover of *Cladonia* spp., in some cases more cover than *Sphagnum* spp. *Spiraea douglasii* Hook., *Malus fusca* (Raf.) C.K. Schneid., *Salix* spp., *Carex obnupta* L.H. Bailey, *C. utriculata* Boot, and *C. aquatilis* Wahlenb. var. *dives* (T. Holm) Kük. are common and abundant species in laggs. Grasses, including invasive *Phalaris arundinacea* L. were not documented in reference laggs but did occur in developed laggs.

Less *Sphagnum* and lichen cover and more feathermoss (e.g., *Pleurozium schreberi*, *Hylocomium splendens*) and deciduous shrub cover in developed peatland center sites is consistent with previously reported vegetation shifts resulting from drier conditions, increased nutrients, fire, or shading (Hebda and Biggs 1981; Gignac et al. 1991; Golinski 2004; Benscoter and Vitt 2008; Ireland and Booth 2012; Pasquet et al. 2015). Road runoff can increase specific ions such as Cl^-^ which decreases *Sphagnum* cover in peatlands (Bubeck et al. 1971; Wilcox 1986; Ehrenfeld and Schneider 1991). Most *Sphagnum* species are not shade tolerant (Clymo and Hayward 1982), so as woody vegetation cover increases, subsequent shading can induce a shift in dominance from *Sphagnum* to feather mosses, which are generally more shade-tolerant (Lachance and Lavoie 2004; Benscoter and Vitt 2008). Feather mosses such as *Pleurozium schreberi* are more drought and shade tolerant than the dominant *Sphagnum* species found in Puget Lowland *Sphagnum*-dominated peatlands (Foster 1985; Gignac et al. 1991; Lachance and Lavoie 2004, Benscoter and Vitt 2008; Pellerin et al. 2016; Laine et al. 2018). In western Canadian continental peatlands, *Pleurozium schreberi* is often a dominant species, especially on the driest hummocks, where it codominates with *Cladonia* species (Gignac and Vitt 1991). Lack of fire may also explain this pattern. Feather mosses (e.g., *Hylocomium splendens*, *Pleurozium schreberi*, etc.) increased in abundance and outcompeted *Sphagnum* spp. in western Canadian boreal bogs that did not experience fire in 80 years (Foster 1985; Benscoter and Vitt 2008). Tribal use of fire is documented in *Sphagnum*-dominated peatlands of the western Olympic peninsula (Anderson 2009) and the cessation of this practice may be resulting in conditions similar to these boreal peatlands. Although documentation of Tribal burning practices in Puget Lowland *Sphagnum*-dominated peatlands is lacking, the incentives for burning these peatlands would be similar to the coastal peatlands.

Tree distribution across structural classes in peatland centers was conspicuously skewed toward short (presumably young) trees. This is consistent with the documented 20th century increase in tree occurrences in *Sphagnum*-dominated peatlands throughout the region (Rocchio et al. 2021) and could be the result of land use stressors, climate change, and/or successional dynamics (Pasquet et al. 2015; Rigg 1917). Increased tree cover has been documented in regional *Sphagnum*-dominated peatlands following disturbance, especially drainage (Golinski 2004; Lachance and Lavoie 2004, Hebda and Biggs 1981). However, the abundance of trees in coastal peatlands of western North America has been noted as a distinguishing characteristic from coastal peatlands of Atlantic Europe (Sjörs 1983; Bisbing et al. 2015).

### Ombrotrophic Status of Puget Lowland Peatlands

Measures of topography, vegetation, hydrology, and pore water chemistry were used to determine whether any study site was functioning as an ombrotrophic peatland (Malmer et al. 1992; Sjörs and Gunnarsson 2002; Tahvanainen 2004; McHaffie et al. 2009; Proctor et al. 2009; Joosten et al. 2017). Evans Creek and Queen’s Bog were the only two study sites that met all ombrotrophic indicators in all seasons. The majority of sites exhibited chemical characteristics of ombrotrophic sites but many lacked evidence of strong downward (precipitation) or upward (groundwater) flow. However, all peatland centers had pH lower than local precipitation and well below local deep groundwater (Turney 1986; Vaccaro et al. 1998; National Atmospheric Deposition Program 2022). Even if groundwater is affecting upper peat profiles, porewater chemistry suggests precipitation is still a predominant water source and likely the strongest factor in the ecology of these sites. The boundary between ombrotrophic and minerotrophic conditions is rarely conspicuous (Proctor et al. 2009). Peatlands with slight inputs of minerotrophic water are flushed during precipitation events, leaving chemical composition very similar to ombrotrophic bogs and have been described as semi-ombrotrophic (Heinselman 1970; Glaser et al. 1990).

Four sites, Arrowhead, Cranberry Marsh #4, Hooven, and Patterson Creek predominantly exhibited indicators of ombrotrophy while 53% (9 study sites) had conflicting indicators of ombrotrophy. Echo Falls, with chemical signatures and summer hydrologic regime indicative of ombrotrophic conditions, may have an ombrotrophic baseline condition, but contemporary stormwater inputs have altered the natural hydrogeochemical regime. Kings Lake also had ombrotrophic chemical signatures, but hydrology measures were inconclusive, with slight downward flow in spring and slight upward flow in summer when pH was also slightly above the 4.5 threshold. This might be explained by winter precipitation counteracting slight upward groundwater flow during the rainy season, but the slightly more minerotrophic water is able to reach into the top 1 m of peat during dry, summer months. However, Kings Lake did have elevated peatland center water tables relative to its lagg, indicative of ombrotrophic conditions. Springer Lake showed a similar hydrological pattern as Kings Lake, but had increased magnesium concentrations in summer, perhaps reflective of the lake water that is likely driving the seasonal upward movement of the water table. The preponderance of evidence for Lower Cedar and Shadow Lake suggests they are not ombrotrophic. The peatland at Shadow Lake likely developed from a process of terrestrialization of a shallow arm of the lake. The peatland’s water table may still be connected to and influenced by lake water chemistry. Shadow Lake is classified as a mesotrophic lake with declining water quality, presumably due to land development in its basin (King County 2021).

Species tolerant of acidic conditions such as ericaceous shrubs and oligotrophic *Sphagnum* spp. dominated peatland centers, while species more commonly found in regional fens, swamps, and marshes dominated the laggs (Golinski 2004; Hebda et al. 2000, Howie et al. 2016; and Rocchio et al. 2021). While these differences are not conclusive evidence of ombrotrophic conditions, they clearly indicate that the peatland centers are more acidic and ion-poor than the laggs, which are more affected by local groundwater, surface water, and overland flow. Minimally, vegetation zonation in our study sites suggests that peatland centers are less affected by these water sources and that precipitation is a primary water source.

Sjörs (1983) noted that a clear hydrological boundary between ombrotrophic bogs and poor fens in southern Alaska and Fennoscandia was difficult to determine. If the ombrotrophic status of each study site was limited to an assessment of chemical indicators, which has been cited as the most objective indicator of ombrotrophic conditions (Proctor et al. 2009), then 88%, or 15 of the study sites would be considered ombrotrophic.

## Conclusions

The *Sphagnum*-dominated peatlands in our study have vegetation and chemical composition similar to ombrotrophic bogs throughout the Northern Hemisphere. Climate, watershed size, and adjacent land use were found to be correlated with differences in ecological characteristics of these peatlands. Effects of land use are likely to manifest first in laggs since they are at the interface of watershed inputs to the peatland basin. It is not unusual to find laggs impacted by adjacent land use stressors, while the interior peatland center remains largely unaffected (Howie and van Meerveld 2011). Hydrological impacts from adjacent land uses were detected in study site laggs but only slight effects were observed in peatland centers. If stressors are persistent and increase over time the lagg may lose its buffering capacity and the peatland center would eventually be exposed to anthropogenic hydrological and chemical impacts (Howie and van Meerveld 2011). These hydrological and water chemistry changes also appear to be affecting vegetation composition in both the peatland centers and laggs.

Integration of the chemical and hydrologic data suggest peatland centers included in this study are supported primarily by precipitation. From a management perspective, a more nuanced view of ombrotrophic conditions is prudent, where recognition of proportional input of precipitation versus groundwater inputs should be the primary consideration, rather than management goals being dictated by a stark line between ombrotrophic and minerotrophic conditions. As demonstrated in this study, the addition of surface and/or groundwater inputs into ombrotrophic, or nearly ombrotrophic peatlands, can result in concerning ecological changes to the site’s ecological functions and biodiversity. Preventing anthropogenically derived surface or groundwater inputs from entering the peatland basin through actions such as avoiding stormwater inputs into the peatland basins, establishing naturally vegetated buffers around the peatland and contributing surface water channels, and keeping the peatland’s watershed with as much natural land cover as possible are recommended for their protection.

## Electronic Supplementary Material

Below is the link to the electronic supplementary material.

Supplementary Material 1

## Data Availability

Selected supporting data are available online from the Washington Department of Natural Resources (DNR) website: https://www.dnr.wa.gov/NHPecoreports. Additional data are available on request from the corresponding author or from DNR, Natural Heritage Program.
