# Supplementary material for: Hydrogeochemical and Vegetation Characterization of Sphagnum-dominated Peatlands in the Puget Lowlands of Washington State, USA
Source: Wetlands (Wilmington). 2025 May 1;45(5):49. doi: 10.1007/s13157-025-01927-7 (PMC12045819; doi:10.1007/s13157-025-01927-7)
Supplement: Supplementary file 1 — Supplementary Material 1 [file 13157_2025_1927_MOESM1_ESM.docx]

**Study Site Locations.**

| **Site Type** | **Site Code** | **Site Name** | **Latitude** | **Longitude** |
| --- | --- | --- | --- | --- |
| Reference | AH | Arrowhead | 47° 11' 48.552" N | 123° 20' 46.1754" W |
| Reference | CM2 | Cranberry Marsh #2 | 47° 11' 3.7674" N | 123° 21' 4.968" W |
| Reference | CM4 | Cranberry Marsh #4 | 47° 11' 33.8274" N | 123° 19' 46.8834" W |
| Reference | KL | Kings Lake | 47° 35' 50.3874" N | 121° 46' 45.3354" W |
| Reference | LD | Lake Dorothy | 47° 47' 3.0474" N | 121° 51' 2.52" W |
| Developed | C12 | Covington 12 | 47° 20' 4.704" N | 122° 1' 0.12" W |
| Developed | C8 | Covington 8 | 47° 20' 14.784" N | 121° 59' 55.716" W |
| Developed | ECH | Echo Falls | 47° 47' 9.672" N | 122° 1' 49.332" W |
| Developed | EC | Evans Creek | 47° 37' 47.82" N | 122° 1' 50.484" W |
| Developed | HO | Hooven | 47° 46' 47.532" N | 122° 7' 23.9874" W |
| Developed | LC | Lower Cedar | 47° 26' 42.576" N | 122° 7' 12.648" W |
| Developed | PA | Patterson Creek | 47° 35' 2.1834" N | 121° 59' 6.864" W |
| Developed | Q | Queen's Bog | 47° 34' 43.284" N | 122° 0' 44.9274" W |
| Developed | SL | Shadow Lake | 47° 24' 22.428" N | 122° 5' 27.8154" W |
| Developed | SPL | Springer Lake | 46° 56' 25.9434" N | 122° 52' 28.6674" W |
| Developed | TR | Trossachs | 47° 35' 52.008" N | 121° 59' 14.928" W |
| Developed | W14 | Wetland 14 | 47° 26' 55.86" N | 122° 5' 28.2834" W |

**Vegetation Synoptic Table**

Color codes: ≥ 80% constancy, 60-79% constancy, 40-59% constancy. Dominant species in **bold** (average ≥ 10% cover when present). Stratum definitions: C=canopy (> 10 m high), SC=subcanopy (5 to 10 m), SH=shrub (0.5 to 5 m), H=herbaceous (< 0.5 m), and G=ground (nonvascular on substrate surface)

|  | | | **Developed Peatland Center**  **(n=12)** | | **Developed Lagg**  **(n=12)** | | **Reference Peatland Center**  **(n=5)** | | **Reference Lagg**  **(n=5)** | |
| --- | --- | --- | --- | --- | --- | --- | --- | --- | --- | --- |
| **Species** | **Growth Form** | **Stratum** | **Const (%)** | **Cover (%)** | **Const. (%)** | **Cover (%)** | **Const. (%)** | **Cover (%)** | **Const. (%)** | **Cover (%)** |
| *Tsuga heterophylla* (Raf.) Sarg. | Tree | C | 17 | 8 | **8** | **38** | 0 | 0 | **40** | **40** |
| *Pinus contorta* Doulas ex Loudon var. *contorta* | Tree | C | 8 | 8 | 0 | 0 | 0 | 0 | 0 | 0 |
| *Pinus monticola* Douglas ex D. Don | Tree | C | 8 | 4 | 0 | 0 | 0 | 0 | 0 | 0 |
| *Thuja plicata* Donn ex D. Don | Tree | C | 0 | 0 | 0 | 0 | 0 | 0 | **40** | **35** |
| *Fraxinus latifolia* Benth. | Tree | C | 0 | 0 | **8** | **18** | 0 | 0 | 0 | 0 |
| *Tsuga heterophylla* (Raf.) Sarg. | Tree | SC | 42 | 6 | 0 | 0 | 20 | 0.1 | 20 | 2 |
| *Pinus contorta* Doulas ex Loudon var. *contorta* | Tree | SC | **8** | **18** | 0 | 0 | 0 | 0 | 0 | 0 |
| *Pinus monticola* Douglas ex D. Don | Tree | SC | 8 | 4 | 0 | 0 | 20 | 0.1 | 0 | 0 |
| *Pseudotsuga menziesii* (Mirb.) Franco | Tree | SC | 8 | 4 | 0 | 0 | 0 | 0 | 0 | 0 |
| *Thuja plicata* Donn ex D. Don | Tree | SC | 8 | 2 | 0 | 0 | 40 | 0.1 | 20 | 8 |
| *Alnus rubra* Bong. | Tree | SC | 0 | 0 | **8** | **38** | 0 | 0 | 0 | 0 |
| *Tsuga heterophylla* (Raf.) Sarg. | Tree | SH | 75 | 6 | 8 | 2 | **60** | **10** | 40 | 2 |
| *Pseudotsuga menziesii* (Mirb.) Franco | Tree | SH | 17 | 1 | 0 | 0 | 0 | 0 | 0 | 0 |
| *Pinus contorta* Doulas ex Loudon var. *contorta* | Tree | SH | 8 | 2 | 0 | 0 | 0 | 0 | 0 | 0 |
| *Pinus monticola* Douglas ex D. Don | Tree | SH | 8 | 4 | 0 | 0 | 20 | 2 | 0 | 0 |
| *Thuja plicata* Donn ex D. Don | Tree | SH | 8 | 0.1 | 0 | 0 | 40 | 1 | 80 | 3 |
| *Picea sitchensis* (Bong.) Carrière | Tree | SH | 0 | 0 | 8 | 4 | 20 | 2 | 0 | 0 |
| *Alnus rubra* Bong. | Tree | SH | 0 | 0 | 17 | 4 | 0 | 0 | 0 | 0 |
| *Tsuga heterophylla* (Raf.) Sarg. | Tree | H | 58 | 1 | 8 | 0.1 | 60 | 2 | 20 | 2 |
| *Pinus contorta* Doulas ex Loudon var. *contorta* | Tree | H | 8 | 0.1 | 0 | 0 | 0 | 0 | 0 | 0 |
| *Pinus monticola* Douglas ex D. Don | Tree | H | 8 | 0.1 | 0 | 0 | 0 | 0 | 0 | 0 |
| *Picea sitchensis* (Bong.) Carrière | Tree | H | 0 | 0 | 0 | 0 | 20 | 0.1 | 0 | 0 |
| *Thuja plicata* Donn ex D. Don *plicata* | Tree | H | 0 | 0 | 8 | 0.1 | 40 | 0.1 | 0 | 0 |
| *Alnus rubra* Bong. | Tree | H | 0 | 0 | 8 | 0.1 | 0 | 0 | 0 | 0 |
| *Rhododendron groenlandicum* (Oeder) Kron & Judd | Shrub | SH | **100** | **50** | 33 | 2 | **100** | **52** | 80 | 2 |
| *Kalmia microphylla* (Hook.) A. Heller | Shrub | SH | **100** | **43** | 17 | 0.1 | **100** | **48** | 20 | 0.1 |
| *Gaultheria shallon* Pursh | Shrub | SH | 17 | 4 | 25 | 7 | 0 | 0 | 60 | 6 |
| *Spiraea douglasii* Hook. | Shrub | SH | 17 | 2 | **75** | **47** | 60 | 1 | **60** | **28** |
| *Frangula purshiana* (de Candolle) A. Gray ssp. *purshiana* | Shrub | SH | 8 | 0.1 | 16 | 5 | 40 | 0.1 | 20 | 8 |
| *Vaccinium corymbosum* L. | Shrub | SH | 8 | 4 | 0 | 0 | 20 | 0.1 | 0 | 0 |
| *Acer circinatum* Pursh | Shrub | SH | 0 | 0 | **17** | **20** | 0 | 0 | 0 | 0 |
| *Cornus occidentalis* (Torr. & A. Gray) Coville | Shrub | SH | 0 | 0 | **17** | **20** | 0 | 0 | 0 | 0 |
| *Lonicera involucrata* (Richardson) Banks ex Spreng. var. *involucrata* | Shrub | SH | 0 | 0 | 0 | 0 | 0 | 0 | 20 | 2 |
| *Malus fusca* (Raf.) C.K. Schneid. | Shrub | SH | 0 | 0 | **67** | **29** | 40 | 2 | **60** | **55** |
| *Rhododendron menziesii* Craven | Shrub | SH | 0 | 0 | 8 | 4 | 0 | 0 | 20 | 2 |
| *Rosa pisocarpa* A. Gray | Shrub | SH | 0 | 0 | 0 | 0 | 0 | 0 | 40 | 4 |
| *Rubus bifrons* Vest | Shrub | SH | 0 | 0 | 8 | 2 | 0 | 0 | 0 | 0 |
| *Rubus laciniatus* Willd. | Shrub | SH | 0 | 0 | 8 | 2 | 0 | 0 | 0 | 0 |
| *Rubus spectabilis* Pursh | Shrub | SH | 0 | 0 | 25 | 4 | 0 | 0 | 20 | 0.1 |
| *Salix geyeriana* Andersson | Shrub | SH | 0 | 0 | 8 | 8 | 0 | 0 | 0 | 0 |
| *Salix hookeriana* Barratt ex Hook. | Shrub | SH | 0 | 0 | 0 | 0 | 0 | 0 | 20 | 8 |
| *Salix scouleriana* Barratt ex Hook. | Shrub | SH | 0 | 0 | **16** | **13** | 0 | 0 | **20** | **68** |
| *Salix sitchensis* Sanson ex Bong. | Shrub | SH | 0 | 0 | 8 | 2 | 0 | 0 | 0 | 0 |
| *Sambucus racemose* L. | Shrub | SH | 0 | 0 | 17 | 2 | 0 | 0 | 0 | 0 |
| *Vaccinium ovalifolium* Sm*.* | Shrub | SH | 0 | 0 | 8 | 4 | 40 | 2 | **40** | **32** |
| *Vaccinium parvifolium* Sm*.* | Shrub | SH | 0 | 0 | 17 | 2 | 0 | 0 | 20 | 0.1 |
| *Vaccinium uliginosum* L. | Shrub | SH | 0 | 0 | 0 | 0 | 20 | 0.1 | 20 | 0.1 |
| *Viburnum edule* (Michx.) Raf. | Shrub | SH | 0 | 0 | 0 | 0 | 0 | 0 | 20 | 0.1 |
| *Vaccinium oxycoccos* L. | Shrub | H | 83 | 2 | 0 | 0 | 60 | 6 | 0 | 0 |
| *Gaultheria shallon* Pursh | Shrub | H | 25 | 6 | 33 | 3 | 60 | 5 | 60 | 6 |
| *Frangula purshiana* (de Candolle) A. Gray ssp. *purshiana* | Shrub | H | 8 | 0.1 | 0 | 0 | 20 | 0.1 | 0 | 0 |
| *Vaccinium ovalifolium* Sm*.* | Shrub | H | 8 | 0.1 | 0 | 0 | 0 | 0 | 0 | 0 |
| *Vaccinium parvifolium* Sm*.* | Shrub | H | 8 | 0.1 | 8 | 0.1 | 0 | 0 | 0 | 0 |
| *Cornus occidentalis* (Torr. & A. Gray) Coville | Shrub | H | 0 | 0 | 8 | 4 | 0 | 0 | 0 | 0 |
| *Lonicera involucrata* (Richardson) Banks ex Spreng. var. *involucrata* | Shrub | H | 0 | 0 | 0 | 0 | 0 | 0 | 20 | 0.1 |
| *Malus fusca* (Raf.) C.K. Schneid. | Shrub | H | 0 | 0 | 17 | 0.1 | 20 | 0.1 | 0 | 0 |
| *Rhododendron groenlandicum* (Oeder) Kron & Judd | Shrub | H | 0 | 0 | 8 | 0.1 | 0 | 0 | 0 | 0 |
| *Rhododendron menziesii* Craven | Shrub | H | 0 | 0 | 0 | 0 | 0 | 0 | 20 | 2 |
| *Rosa pisocarpa* A. Gray | Shrub | H | 0 | 0 | 0 | 0 | 0 | 0 | 20 | 0.1 |
| *Spiraea douglasii* Hook. | Shrub | H | 0 | 0 | 17 | 1 | 20 | 0.1 | 0 | 0 |
| *Vaccinium uliginosum* L. | Shrub | H | 0 | 0 | 0 | 0 | 40 | 0.1 | 0 | 0 |
| *Rubus ursinus* Cham. & Schltdl. | Shrub | H | 0 | 0 | 17 | 0.1 | 0 | 0 | 20 | 0.1 |
| *Pteridium aquilinum* (L.) Kuhn ssp. *pubescens* (Underw.) Piper & Beattie | Herb | H | 17 | 4 | 17 | 4 | 60 | 1 | 60 | 3 |
| *Carex utriculata* Boot | Herb | H | 17 | 1 | 8 | 0.1 | 60 | 1 | 20 | 2 |
| *Drosera rotundifolia* L. | Herb | H | 8 | 0.1 | 0 | 0 | 20 | 0.1 | 0 | 0 |
| *Lysichiton americanus* Hulten & H. St. John | Herb | H | 8 | 4 | 42 | 8 | 40 | 0.1 | 60 | 2 |
| *Eriophorum chamissonis* C.A. Mey. | Herb | H | 8 | 0.1 | 0 | 0 | 20 | 2 | 0 | 0 |
| *Juncus bufonius* L. | Herb | H | 8 | 0.1 | 0 | 0 | 0 | 0 | 0 | 0 |
| *Athyrium filix-femina* (L.) Roth ex Mertens ssp. *cyclosorum* (Rupr.) C. Chr. | Herb | H | 0 | 0 | 42 | 2 | 0 | 0 | 20 | 0.1 |
| *Dryopteris expansa* (C. Presl) Fraser-Jenk. & Jermy | Herb | H | 0 | 0 | 8 | 0.1 | 0 | 0 | 0 | 0 |
| *Polystichum munitum* (Kaulf.) C. Presl | Herb | H | 0 | 0 | 33 | 1 | 0 | 0 | 20 | 0.1 |
| *Struthiopteris spicant* (L.) Weiss | Herb | H | 0 | 0 | 8 | 0.1 | 40 | 0.1 | 40 | 0.1 |
| *Callitriche stagnalis* Scop. | Herb | H | 0 | 0 | 8 | 4 | 0 | 0 | 0 | 0 |
| *Comarum palustre* L. | Herb | H | 0 | 0 | 8 | 0.1 | 0 | 0 | 0 | 0 |
| *Cornus unalaschkensis* Ledeb. | Herb | H | 0 | 0 | 0 | 0 | 40 | 1 | 40 | 1 |
| *Dicentra formosa* (Haw.) Walp. ssp. *formosa* | Herb | H | 0 | 0 | 17 | 0.1 | 0 | 0 | 0 | 0 |
| *Epilobium* spp. | Herb | H | 0 | 0 | 8 | 0.1 | 0 | 0 | 0 | 0 |
| *Galium* spp. | Herb | H | 0 | 0 | 8 | 0.1 | 0 | 0 | 0 | 0 |
| *Gentiana sceptrum* Griseb. | Herb | H | 0 | 0 | 0 | 0 | 0 | 0 | 20 | 0.1 |
| *Geranium robertianum* L. | Herb | H | 0 | 0 | 8 | 2 | 0 | 0 | 0 | 0 |
| *Hippuris vulgaris* L. | Herb | H | 0 | 0 | 8 | 2 | 0 | 0 | 0 | 0 |
| *Iris pseudacorus* L. | Herb | H | 0 | 0 | 8 | 0.1 | 0 | 0 | 0 | 0 |
| *Lemna minor* L. | Herb | H | 0 | 0 | 8 | 2 | 0 | 0 | 0 | 0 |
| *Lemna* spp. | Herb | H | 0 | 0 | 17 | 0.1 | 0 | 0 | 0 | 0 |
| *Ludwigia palustris* (L.) Elliott | Herb | H | 0 | 0 | 17 | 1 | 0 | 0 | 0 | 0 |
| *Lycopus* spp. | Herb | H | 0 | 0 | 8 | 0.1 | 0 | 0 | 0 | 0 |
| *Lycopus uniflorus* Michx. | Herb | H | 0 | 0 | 8 | 0.1 | 0 | 0 | 0 | 0 |
| *Lysimachia europaea* (L.) U. Manns & Anderb. | Herb | H | 0 | 0 | 8 | 0.1 | 40 | 0.1 | 20 | 0.1 |
| *Maianthemum dilatatum* (Alph. Wood) A. Nelson & J.F. Macbr. | Herb | H | 0 | 0 | 0 | 0 | 20 | 8 | 20 | 2 |
| *Mycelis muralis* (L.) Dumort. | Herb | H | 0 | 0 | 17 | 0.1 | 0 | 0 | 0 | 0 |
| *Myriophyllum* spp. | Herb | H | 0 | 0 | 17 | 1 | 0 | 0 | 0 | 0 |
| *Nuphar polysepala* Engelm. | Herb | H | 0 | 0 | **8** | **18** | 0 | 0 | 0 | 0 |
| *Oenanthe sarmentosa* C. Presl ex DC. | Herb | H | 0 | 0 | 33 | 8 | 0 | 0 | 20 | 0.1 |
| *Persicaria lapathifolia* (L.) Gray | Herb | H | 0 | 0 | 8 | 4 | 0 | 0 | 0 | 0 |
| *Persicaria* spp. | Herb | H | 0 | 0 | 8 | 0.1 | 0 | 0 | 0 | 0 |
| *Potamogeton* spp. | Herb | H | 0 | 0 | 8 | 2 | 0 | 0 | 0 | 0 |
| *Ranunculus repens* L. | Herb | H | 0 | 0 | 8 | 0.1 | 0 | 0 | 0 | 0 |
| *Rumex* spp. | Herb | H | 0 | 0 | 8 | 0.1 | 0 | 0 | 0 | 0 |
| *Solanum dulcamara* L. | Herb | H | 0 | 0 | 33 | 1 | 0 | 0 | 0 | 0 |
| *Veronica americana* Schwein. ex Benth. | Herb | H | 0 | 0 | 8 | 0.1 | 0 | 0 | 0 | 0 |
| *Veronica scutellate* L. | Herb | H | 0 | 0 | 17 | 2 | 0 | 0 | 0 | 0 |
| *Xerophyllum tenax* (Pursh) Nutt. | Herb | H | 0 | 0 | 0 | 0 | **20** | **38** | 0 | 0 |
| *Agrostis scabra* Willd. | Herb | H | 0 | 0 | 8 | 0.1 | 0 | 0 | 0 | 0 |
| *Glyceria elata* (Nash) M.E. Jones | Herb | H | 0 | 0 | 8 | 4 | 0 | 0 | 0 | 0 |
| *Phalaris arundinacea* L. | Herb | H | 0 | 0 | 17 | 4 | 0 | 0 | 0 | 0 |
| *Torreyochloa pallida* (Torr.) G.L. Church var. *pauciflora* (J. Presl) J.I. Davis | Herb | H | 0 | 0 | 8 | 0.1 | 0 | 0 | 0 | 0 |
| *Carex aquatilis* Wahlenb. var. *dives* (T. Holm) Kük. | Herb | H | 0 | 0 | 8 | 0.1 | 0 | 0 | 0 | 0 |
| *Carex arcta* Boott | Herb | H | 0 | 0 | 8 | 2 | 0 | 0 | 0 | 0 |
| *Carex canescens* L. | Herb | H | 0 | 0 | 25 | 0.1 | 0 | 0 | 0 | 0 |
| *Carex cusickii* Mack. ex Piper & Beattie | Herb | H | 0 | 0 | 17 | 2 | 0 | 0 | 0 | 0 |
| *Carex echinata* Murray ssp. *echinata* | Herb | H | 0 | 0 | 8 | 0.1 | 0 | 0 | 0 | 0 |
| *Carex leptopoda* Mack. | Herb | H | 0 | 0 | 17 | 0.1 | 0 | 0 | 0 | 0 |
| *Carex obnupta* L.H. Bailey | Herb | H | 0 | 0 | 0 | 0 | 0 | 0 | **60** | **72** |
| *Eleocharis* spp. | Herb | H | 0 | 0 | 8 | 0.1 | 0 | 0 | 0 | 0 |
| *Juncus acuminatus* Michx. | Herb | H | 0 | 0 | 8 | 0.1 | 0 | 0 | 0 | 0 |
| *Juncus balticus* Willd. | Herb | H | 0 | 0 | 8 | 8 | 0 | 0 | 0 | 0 |
| *Juncus hesperius* (Piper) Lint | Herb | H | 0 | 0 | 17 | 0.1 | 0 | 0 | 0 | 0 |
| *Juncus* spp. | Herb | H | 0 | 0 | 8 | 0.1 | 0 | 0 | 0 | 0 |
| *Rhynchospora alba* (L.) Vahl. | Herb | H | 0 | 0 | 0 | 0 | 20 | 2 | 0 | 0 |
| *Scirpus cyperinus* (L.) Kunth | Herb | H | 0 | 0 | 8 | 4 | 0 | 0 | 0 | 0 |
| *Scirpus microcarpus* J. Presl & C. Presl | Herb | H | 0 | 0 | 8 | 0.1 | 0 | 0 | 0 | 0 |
| *Sparganium emersum* Rehmann | Herb | H | 0 | 0 | 17 | 4 | 0 | 0 | 0 | 0 |
| *Typha latifolia* L. | Herb | H | 0 | 0 | **8** | **18** | 0 | 0 | 0 | 0 |
| *Hedera helix* L. | Herb | H | 0 | 0 | 8 | 4 | 0 | 0 | 0 | 0 |
| *Sphagnum* spp. | Nonvascular | G | **100** | **26** | 33 | 7 | **100** | **48** | 40 | 2 |
| Undifferentiated Feather Moss | Nonvascular | G | **92** | **42** | 25 | 2 | **80** | **19** | **60** | **42** |
| Undifferentiated Brown Moss | Nonvascular | G | 58 | 4 | 8 | 2 | 20 | 0.1 | 0 | 0 |
| Undifferentiated Lichen | Nonvascular | G | **42** | **13** | 0 | 0 | **100** | **13** | 0 | 0 |
| Undifferentiated Moss (non-*Sphagnum*, non-Feather) | Nonvascular | G | 33 | 4 | 25 | 2 | 40 | 2 | 0 | 0 |
| *Ricciocarpos natans* (L.) Corda | Nonvascular | G | 0 | 0 | 8 | 0.1 | 0 | 0 | 0 | 0 |
| *Polytrichum commune* Hedw. | Nonvascular | G | 0 | 0 | 8 | 0.1 | 0 | 0 | 0 | 0 |
| Bare soil | n/a | G | **25** | **11** | 25 | 2 | 20 | 0.1 | 0 | 0 |
| Litter | n/a | G | 8 | 8 | 33 | 9 | 0 | 0 | **20** | **38** |
| Water | n/a | G | 0 | 0 | **42** | **25** | 0 | 0 | 0 | 0 |
